# Transcriptome-wide analysis of trigeminal ganglion and subnucleus caudalis in a mouse model of chronic constriction injury-induced trigeminal neuralgia

**DOI:** 10.3389/fphar.2023.1230633

**Published:** 2023-09-28

**Authors:** Xiaona Cui, Bo Qin, Chaoyun Xia, Hong Li, Zhiye Li, Zhisong Li, Abdul Nasir, Qian Bai

**Affiliations:** ^1^ Medical Research Center, The Second Affiliated Hospital of Zhengzhou University, Zhengzhou, Henan, China; ^2^ Department of Anesthesiology and Perioperative Medicine, The Second Affiliated Hospital of Zhengzhou University, Zhengzhou, Henan, China; ^3^ Department of Anesthesiology, International Peace Maternity & Child Health Hospital, Shanghai Jiaotong University, School of Medicine, Shanghai, China; ^4^ Translational Medical Center, The First Affiliated Hospital of Zhengzhou University, Zhengzhou, Henan, China; ^5^ Department of Pharmacy, The Second Affiliated Hospital of Zhengzhou University, Zhengzhou, Henan, China

**Keywords:** neuropathic pain, allodynia, hyperalgesia, differentially expressed genes, RNA sequencing, non-coding RNAs

## Abstract

Trigeminal neuropathic pain (TNP) induces mechanical allodynia and hyperalgesia, which are known to alter gene expression in injured dorsal root ganglia primary sensory neurons. Non-coding RNAs (ncRNAs) have been linked to TNP. However, the functional mechanism underlying TNP and the expression profile of ncRNAs in the trigeminal ganglion (TG) and trigeminal subnucleus caudalis (Sp5C) are still unknown. We used RNA sequencing and bioinformatics analysis to examine the TG and Sp5C transcriptomes after infraorbital nerve chronic constrictive injury (IoN-CCI). The robust changes in the gene expression of lncRNAs, circRNAs, and mRNAs were observed within the TG and Sp5C from mice that underwent IoN-CCI and the sham-operated mice (day 7). In total, 111,003 lncRNAs were found in TG and 107,157 in Sp5C; 369 lncRNAs were differentially expressed in TG, and 279 lncRNAs were differentially expressed in Sp5C. In addition, 13,216 circRNAs in TG and 21,658 circRNAs in Sp5C were identified, with 1,155 circRNAs and 2,097 circRNAs differentially expressed in TG and Sp5C, respectively. Furthermore, 5,205 DE mRNAs in TG and 3,934 DE mRNAs in Sp5C were differentially expressed between IoN-CCI and sham groups. The study revealed a high correlation of pain-related differentially expressed genes in the TG and Sp5C to anxiety, depression, inflammation, neuroinflammation, and apoptosis. Gene Ontology analysis revealed that binding-related molecular functions and membrane-related cell components were significantly enriched. Kyoto Encyclopedia of Genes and Genomes analysis shows the most significant enrichments in neurogenesis, nervous system development, neuron differentiation, adrenergic signaling, cAMP signaling, MAPK signaling, and PI3K-Akt signaling pathways. Furthermore, protein–protein interaction analysis showed that hub genes were implicated in neuropeptide signaling pathways. Functional analysis of DE ncRNA-targeting genes was mostly enriched with nociception-related signaling pathways underpinning TNP. Our findings suggest that ncRNAs are involved in TNP development and open new avenues for research and treatment.

## 1 Introduction

Trigeminal neuropathic pain (TNP) or trigeminal neuralgia is a rare chronic neuropathic pain (NP) condition. The pain is often described as a stabbing, shooting, or electric shock-like sensation that can last from seconds to minutes caused by damage or injury of one or more nerve roots of the trigeminal ganglion (Goadsby and Evers, 2020; Orofacial, 2020). Due to its severity, recurrence, and trigger point, it has long been extensively studied by the medical community (Mueller et al., 2011; Zakrzewska and McMillan, 2011). It can be caused by trigeminal neuralgia, nerve trauma, and demyelinating disorders (Bista and Imlach, 2019). Chronic pain goes beyond physical effects, deeply impacting emotions and bridging physiological and psychological domains. Emotional disorders (anxiety, depression, and stress) entwine with diverse pain syndromes, magnifying pain perception and fostering a bidirectional link, where chronic pain fuels emotional issues (Keefe et al., 2001; Lumley et al., 2011; Gilam et al., 2020). This highlights the necessity of holistic understanding for diagnosis and treatment. However, despite the efforts made to understand the underlying pathophysiological mechanisms of TNP, they are still poorly understood (Benoliel et al., 2012). In clinical practice, several methods are used to manage TNP, including sodium channel blocker drug treatment (carbamazepine and oxcarbazepine) and surgical intervention (microvascular decompression, ethanol injection, radiofrequency thermocoagulation, balloon inflation mechanical compression, and stereotactic radiotherapy) (Cruccu, 2017), but they are not specific to TNP and often result in ineffective pain relief (Christoforou, 2018) and unwanted side effects. Although numerous molecular targets in the trigeminal ganglion (TG) and trigeminal subnucleus caudalis (Sp5C) have been identified for the treatment of TNP, no consistently effective treatment options are currently available (Nagakura et al., 2021). Preclinical research is needed to understand TNP’s molecular mechanism and find new therapeutic targets to improve TNP treatment.

In recent years, ncRNAs have been shown to regulate gene expression under pathological and physiological conditions in NP (Cai et al., 2020; Kalpachidou et al., 2020; Pan et al., 2021; Du et al., 2022; Bai et al., 2023). Non-coding ribose nucleic acids (ncRNAs) are RNA molecules that do not encode proteins but function to regulate gene expression. ncRNAs are classified into four types based on their size: microRNAs (miRNAs, 18–23 nt), long ncRNAs (lncRNAs, >200 nt), circular RNAs (circRNAs, >100 nt), and small nucleolar RNAs (snoRNAs). Several chronic disorders reveal unique miRNA and lncRNA expression signatures, which recently generated big hopes for new perspectives for the development of diagnostic applications. ncRNAs modulating both neuronal and immune processes further promise therapeutic potential for diseases with a neuro-immune component (ref). Few studies have highlighted the key role of miRNAs in the TG in orofacial inflammatory pain and oral cancer pain (Dong et al., 2014; Pereira et al., 2017). Additionally, lncRNAs alongside circRNAs and miRNAs all perform a critical function in controlling mRNA expression during NP progression (Zhang et al., 2019; Song et al., 2020). Notably, recent research has shown that lncRNAs in the TG also contribute to chronic inflammatory temporomandibular joint pain and TNP (Xu et al., 2020; Dong et al., 2022). Despite this knowledge, the regulatory functions and mechanisms of ncRNAs in the TG and Sp5C under TNP have not been systematically described. Although studies on the differential gene expression changes in the dorsal root ganglia *versus* the TG in response to peripheral nerve injury have been conducted (Korczeniewska et al., 2020; Xu et al., 2022), the genome-wide expression and functional roles of ncRNAs in different regions related to TNP are not well understood. Therefore, there is a pressing need to identify the gene interactions and gene expression profiles in pain-associated regions to understand TNP pathogenesis better and develop novel therapeutic strategies.

This study used a whole-transcriptome sequencing approach to comprehensively examine ncRNA and mRNA expression signatures in the TG and Sp5C of infraorbital nerve chronic constrictive injury (IoN-CCI)-induced TMP mice. Furthermore, bioinformatics analysis was used to interpret the potential biological functions of pain-related differentially expressed (DE) genes, DE ncRNAs and DE mRNAs, and the potential regulatory mechanisms of ncRNAs in TNP.

## 2 Materials and methods

### 2.1 Animal preparation

Seven-week-old male C57BL/6 mice weighing approximately 20–23 g were purchased from the Zhengzhou Animal Experiment Center (Henan, China). The mice were kept in standard cages with a 12-h light/dark cycle at 23°C ± 1°C and fed with a standard laboratory diet and tap water *ad libitum*. Before the experimentation, the mice were acclimatized for 7 days. All procedures adhered to the guidelines established by the International Association for the Study of Pain and were approved by the Zhengzhou University Animal Care and Use Committee.

### 2.2 Trigeminal neuropathic pain mouse model

To induce TNP in mice, we conducted an IoN-CCI model, as previously described (Kim et al., 2014; Liu S. et al., 2020). The IoN-CCI model for NP is widely accepted (Kernisant et al., 2008), as IoN has no autonomic components. Then, mice were anesthetized with 4% isoflurane, and a 4-mm-long incision was made along the gingivobuccal margin, proximal to the first molar. The left IoN was carefully separated from the adhering tissues, and two ligatures (5–0 chromic gut) were tied at 1 mm intervals around the nerve. Using a 3M tissue adhesive, the incision was closed. The nerves of sham mice were merely exposed; no ligation was performed. All surgical procedures were performed under aseptic conditions, with no antibiotics used.

### 2.3 Behavioral test

#### 2.3.1 Mechanical hypersensitivity test

To assess orofacial mechanical hypersensitivity, mice were placed in a 10-cm-long restraining Plexiglas cylinder where they could extend their heads and forepaws but not turn around. We used von Frey filaments with increasing strengths from 0.07, 0.16, 0.4, 0.6, 1.0, and 1.4 up to 2.0 g to measure the head withdrawal threshold (HWT) of skin areas innervated by trigeminal nerve V2 and V3 branches on days 3 and 7. Each filament was applied five times with a 10-s interval to the innervated skin area for 1–2 s, after a 5-min acclimation period. A positive response was noted when the mouse sharply withdrew its head in response to the stimulation (Bai et al., 2019).

#### 2.3.2 Cold allodynia test

Mice were placed in plexiglass boxes (7 × 9 × 11 cm) on elevated wire mesh platforms and allowed to acclimate for 30 min. We administered 20 uL acetone to the left vibrissal pad skin surface (ipsilateral to surgery) and recorded grooming time over 60 s. Three times, 10 min apart, acetone was put on the face, and the average time spent rubbing/scratching the face was recorded. Nociceptive time was measured after acetone exposure and compared to the baseline (intra-animal) or sham-operated (inter-animal) values to ascertain the presence or absence of cold allodynia (Ma et al., 2012; Trevisan et al., 2016).

### 2.4 Tissue collection and RNA extraction

Unilateral punches of TG and Sp5C were used to collect tissues from both the experimental and sham groups of mice on day 7. For each sample, we combined the punches from three different mice, yielding nine animals for each treatment group. Total RNA was extracted using TRIzol (Takara Biomedical Technology, Beijing, China) and purified using the RNeasy Micro Kit 50 (cat. 74004, Qiagen). Utilizing a NanoDrop 2000 spectrophotometer (Thermo NanoDrop One, Beijing, China), the concentration of purified RNA was determined. The sample’s quality was assessed using the A_260_/A_280_ (1.97–2.08) and RNA integrity numbers (RINs) ranging from 7.5 to 8.4 values.

### 2.5 RNA sequencing

The extracted RNA underwent rRNA depletion using the Ribo-Zero rRNA Removal (Human/Mouse/Rat) Kit (Illumina, San Diego, CA, United States). Afterward, using the TruSeq Stranded Total RNA Sample Preparation Kit (Illumina), RNA libraries were generated in a strand-specific manner without poly-A selection, following the manufacturer’s protocols. Subsequently, RNA sequencing was performed on the Illumina Nova 6,000 Plate High Output Model (Illumina, San Diego, United States) with over 1,087 M reads per lane.

### 2.6 Bioinformatics analysis

The TG and Sp5C samples were analyzed for differential gene expression using transcript, lncRNA and circRNA, and expression analysis. The sequences were quality-checked with Trimmomatic 0.32 before being mapped to the *musculus* genome sequence (version GRCm38.72). Gene hit counts and RPKM were calculated using CLCbio software (CLC Genomics Workbench 7.0.2, CLC Genomics Server). Using bigWig files derived from bam files, the mapped reads were visualized in the UCSC browser. Significant DE mRNAs were defined by utilizing a cutoff of *p* ≤ 0.05 and fold change ≥ 2 for subsequent analyses, such as Gene Ontology (GO) term and Kyoto Encyclopedia of Genes and Genomes (KEGG) pathway analysis, to derive more relevant information, particularly for functional analysis and comparisons between the two distinct regions. Heatmaps were generated with OmicShare (GENE DENOVO), and the Comparative Toxicogenomics and GeneCards databases were used to analyze the functions of DE mRNAs. Using Venn diagrams, we mapped DE mRNAs to pain-related genes in two distinct regions and compared differentially expressed genes (DEGs) to genes involved in neuroinflammation (inflammation and immunity) and apoptosis.

### 2.7 Functional enrichment analysis of differentially expressed genes

For the function analysis, more than 5,202 and 3,934 DE mRNAs (*p <* 0.05, fold change ≥ 2) from TG and Sp5C, respectively, were classified using the KEGG and GO analyses by the Database for Annotation, Visualization, and Integrated Discovery (DAVID5) (Li et al., 2020; Su et al., 2021). In addition, GO annotations and KEGG pathway analyses were applied to predict the function of DE lncRNAs (Liu et al., 2022).

### 2.8 Real-time quantitative polymerase chain reaction

The RNA sequence analysis results were verified through q-PCR. A SteadyPure Quick RNA Extraction Kit (Accurate Biotechnology, Hunan, China) was used to extract total RNA, and then, the Evo M-MLV RT Mix Kit with gDNA Clean for qPCR (also from Accurate Biotechnology) was used to reverse transcribe the RNA into cDNA for quantitative polymerase chain reaction. A 2 µL template was then amplified by PCR (Servicebio, Wuhan, China) using primers (Supplementary Table S1) in a reaction volume of 20 μL, including 250 nM of each primer (forward and reverse), 10 µL of the SYBR Green Premix Pro Taq HS qPCR Kit (Rox Plus) (Accurate Biotechnology, Hunan, China), and 20 ng of cDNA. The reactions were performed using a 7,500 Fast Real-Time PCR Detection System (Applied Biosystems, United States) under the following conditions: 95°C for 30 s followed by 40 cycles of 95°C for 5 s and 60°C for 30 s. The ratios (IoN-CCI mRNA levels to sham mRNA levels) were calculated using the Ct method (2^−ΔΔCT^) by normalizing all data to the housekeeping gene glyceraldehyde-3-phosphate dehydrogenase (GAPDH).

### 2.9 Protein–protein interaction network construction

The functional connection between the DEGs from the two regions was analyzed and elucidated using the STRING (Search Tool for the Retrieval of Interacting Genes) version: 11.0 database. This study selected the top 50 DEGs with the highest correlation degree for each region and used the Cytoscape program (version 3.6.0) to construct a network (Su et al., 2021). The nodes in the network represent genes, and the edges represent functional interactions between them. Using the cityscape plugin, the connection degree of each node was computed, and the node size was established depending on the connection degree. The nodes were color-coded to indicate whether the gene was upregulated (red) or downregulated (blue) in the analyzed samples.

### 2.10 CeRNA network construction

The miRNA–lncRNA–mRNA and miRNA–circRNA–mRNA networks were established in this study. To conduct the ceRNA network analysis for the miRNA–lncRNA–mRNA ceRNA and miRNA–circRNA–mRNA ceRNA networks, we analyzed DE miRNAs and their target mRNAs and lncRNAs or circRNA. We calculated the Pearson correlation coefficient and significant *p*-value between mRNA and lncRNAs or circRNA expression. For comparing two sample groups without duplication, we determined the *p*-value of the hypergeometric test for the number of miRNAs combined with lncRNAs or circRNA. The ceRNA regulatory network was constructed based on the same direction of differential expression of DE mRNAs and lncRNAs or circRNA. For multiple groups or repeated samples, we constructed the ceRNA regulatory network based on PPC ≥ 0.5 and *p*-value ≤ 0.05. The number of miRNAs linked by mRNA–lncRNA or mRNA–circRNA pairs, the Pearson coefficient of DE or expression level correlation, and the *p*-value were displayed along with the top two miRNAs and their target genes in accordance with their respective network degrees of mRNA and lncRNA or circRNA.

### 2.11 Statistical analysis

The data collected in this study were presented as mean ± SEM and obtained randomly. The data were statistically analyzed with a two-tailed, unpaired Student’s t-test and one-way and two-way ANOVA with repeated measures. The *post hoc* Tukey method was employed for pairwise comparisons when ANOVA showed significant differences. A *p*-value of *<* 0.05 was considered statistically significant. GraphPad Prism 8.0 was used to analyze data.

## 3 Result

### 3.1 IoN-CCI leads to nociceptive hypersensitivities

**FIGURE 1:**
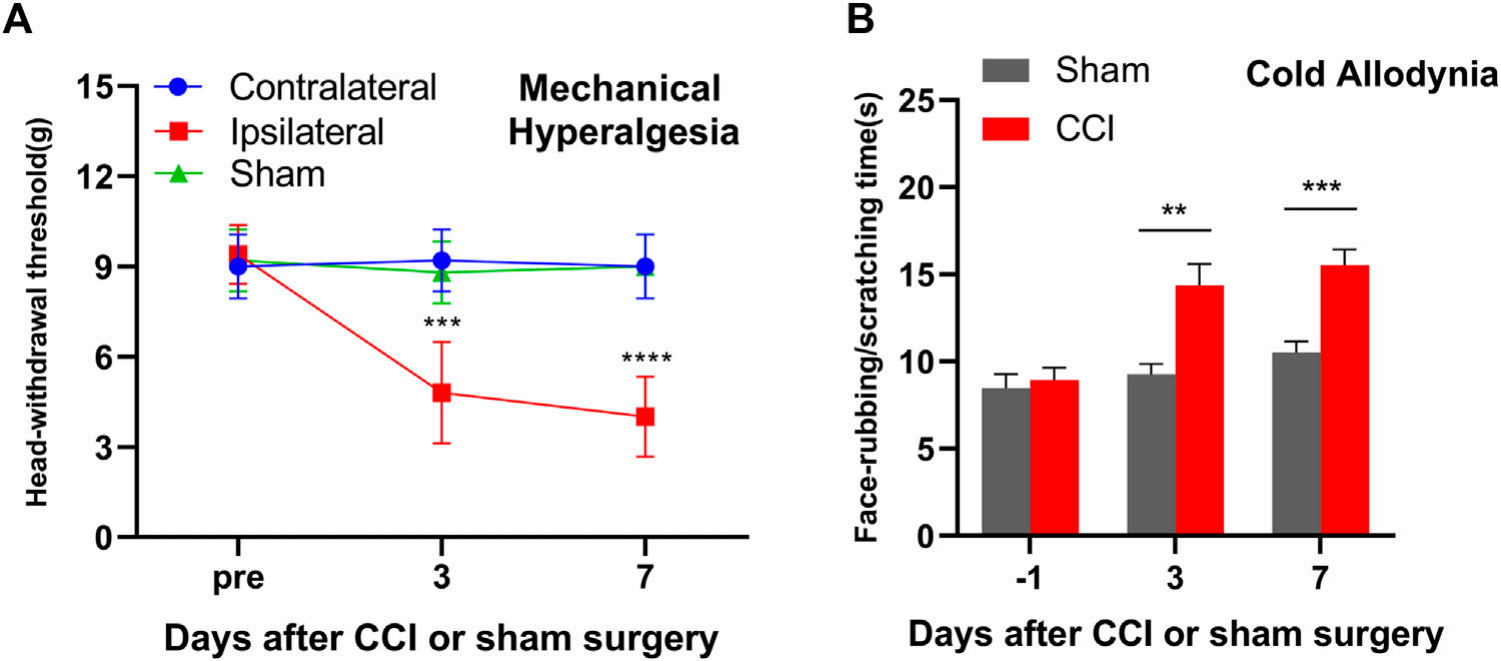
Mice with unilateral infraorbital nerve chronic constrictive injury (IoN-CCI) developed nociceptive hypersensitivities. **(A)** Head withdrawal threshold in response to von Frey filament in ascending order stimuli on the ipsilateral, contralateral, and sham on days 3 and 7 after IoN-CCI or sham surgery. **(B)** Face rubbing/scratching time in response to cold stimuli on the ipsilateral side after IoN-CCI or sham surgery on days 3 and 7. n = 10/group. Two-way ANOVA with repeated measures and the *post hoc* Tukey test showed ***p < 0.001 and ****p < 0.0001 *versus* the sham group or contralateral side.

The IoN-CCI surgery mice displayed a significant mechanical hyperalgesia on days 3 and 7 as indicated by the decrease in the HWT in response to von Frey filament on the ipsilateral facial side (*p* < 0.0001) as compared to the contralateral facial side (*p* > 0.05) (Figure 1A). On the other hand, the sham control mice exhibited a similar pattern of the baseline mechanical threshold range in both the contralateral and ipsilateral facial sides thought out treatment from day 0 to day 7 (Figure 1C). These results are parallel with previous reports on mice exposed to IoN-CCI (Dieb and Hafidi, 2013; Kim et al., 2014; Trevisan et al., 2016; Bai et al., 2019). Additionally, the mice with IoN-CCI surgery displayed a significant increase in cold allodynia, as evidenced by an increase in the duration of rubbing/scratching compared to the sham control mice on day 3 (*p <* 0.01) and day 7 (*p <* 0.001), as depicted in Figure 1B.

### 3.2 RNA-seq and genome-wide read mapping in the TG and Sp5C after IoN-CCI

The RNA-seq resulted in over 97 million (M) reads in each group, including 99.88 M in sham TG, 104.38 M in IoN-CCI group TG, 97.48 M in sham Sp5C, and 111.42 M in IoN-CCI group Sp5C. Initially, a total of 1,239,516,814 raw reads were produced from the Illumina HiSeq 6000 platform. However, after applying quality control steps, such as removing the adaptor sequences and low-quality sequences, a total of 1,210,555,880 clean reads with a range of 97.50%–98.08% were obtained. After the reads were trimmed, they were mapped to the ENSEMBLE reference mouse genome (GRCm38.90) and then sorted into exonic, intronic, and intergenic regions. Each group’s share of mapped reads in the TG and Sp5C regions was depicted as a percentage. In both the sham and CCI groups, high proportions of readings were aligned to exonic regions, followed by a sizeable number of reads mapped to intronic areas (Supplementary Figure S1A). The IoN-CCI procedure significantly increased the expression of the intronic and intergenic regions of both areas of the brain, which could influence altered gene expression underlying TNP, as shown in Supplementary Figures S1B–C.

We then analyzed the DEG expression profiles in TG and Sp5C, and approximately 1,134 and 648 significant genes were identified out of a total of 41,958 and 40,280 transcripts, respectively, in TG and Sp5C 7 days after CCI. Protein-coding RNAs had the largest transcriptional changes (75.98%–76.56%), followed by other ncRNAs (13.83%–14.12%), known lncRNAs (9.27%–9.55%), and predicted lncRNAs (0.34%–0.35%) in both regions (Supplementary Figures S1D–E). The transcript length distribution in mRNAs and lncRNAs and the number of exons in mRNAs and lncRNAs are shown in Supplementary Figure S1F, which compares the genomic architecture of new predicted lncRNAs, known lncRNAs, and mRNAs.

### 3.3 IoN-CCI modulates the expression of ncRNAs and mRNAs in the mice TG and Sp5C

**FIGURE 2:**
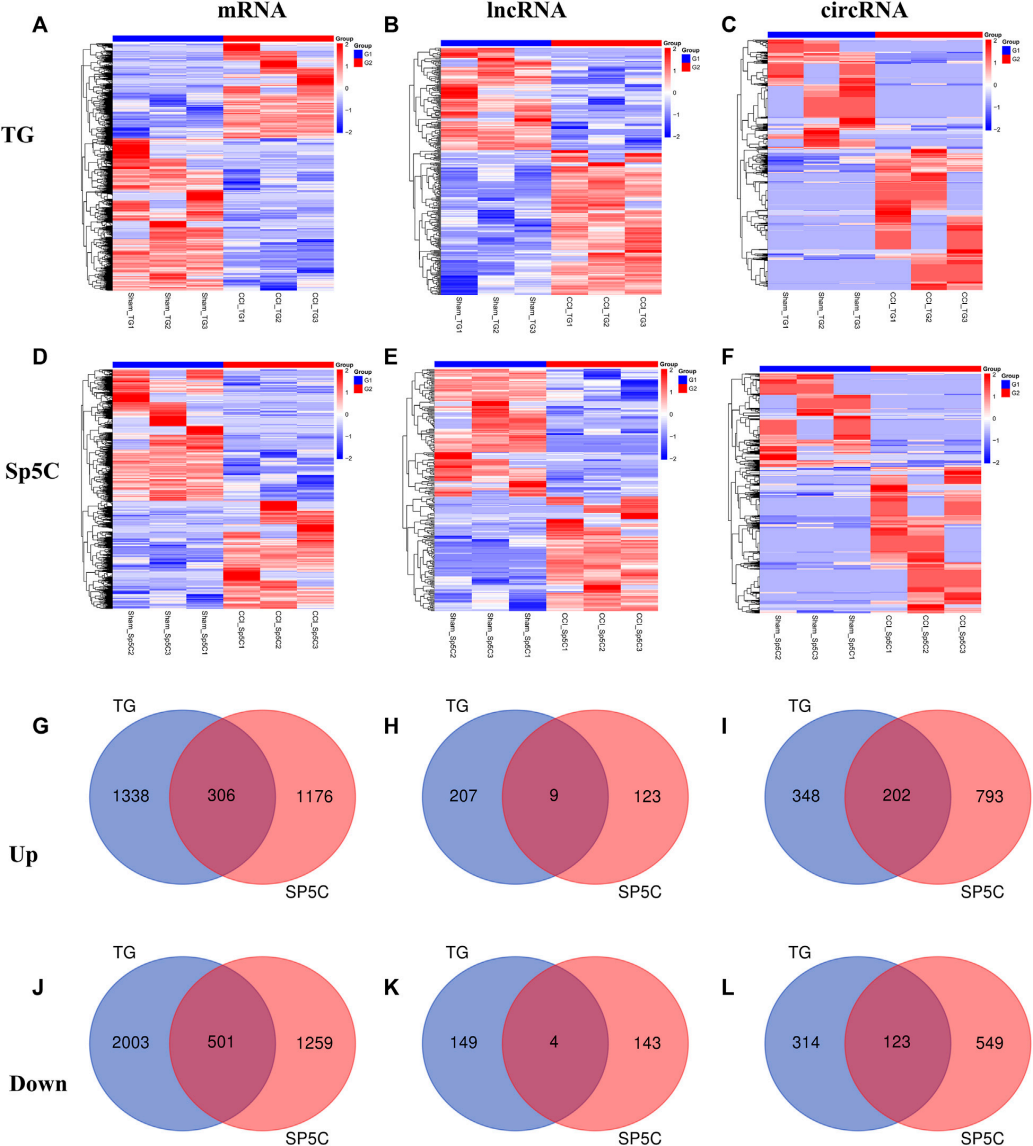
Differentially expressed mRNAs, lncRNAs, and circRNAs in the TG and Sp5C after nerve injury. **(A–F)** Heatmaps of significantly differentially expressed mRNAs **(A, D)**, lncRNAs **(B, E)**, and circRNAs **(C, F)** in the TG and Sp5C from mice on day 7 after CCI-ION or sham surgery. n = 9 mice/group. **(G, J)** Venn diagrams indicate the co-upregulated mRNAs **(G)** and co-downregulated mRNAs **(J)** in the TG and Sp5C on day 7 after surgery. n = 9 mice/group. **(H, K)** Venn diagrams indicate the co-upregulated lncRNAs **(H)** and co-downregulated lncRNAs **(K)** in the TG and Sp5C on day 7 after surgery. n = 9 mice/group. (I, L) Venn diagrams indicate the co-upregulated CircRNAs **(I)** and co-downregulated CircRNAs **(L)** in the TG and Sp5C on day 7 after surgery. n = 9 mice/group.

After IoN-CCI, the TG and Sp5C showed significant changes in mRNA, lncRNA, and circRNA gene expression. In the TG, approximately 5,202 mRNAs (1,997 upregulated and 3,205 downregulated), 369 lncRNAs (216 upregulated and 153 downregulated), and 1,155 circRNAs (651 upregulated and 504 downregulated) were significantly altered, while in the Sp5C, approximately 3,933 mRNAs (1,776 upregulated and 2,157 downregulated), 279 lncRNAs (132 upregulated and 147 downregulated), and 2,097 circRNAs (1,280 upregulated and 817 downregulated) were significantly altered (Supplementary Figures S2A–F). The clustered heatmaps of DE mRNAs (Figures 2A,D), DE lncRNAs (Figures 2B,E), and DE circRNAs (Figures 2C,F) revealed distinct gene expression patterns across TG and Sp5C after IoN-CCI. Venn diagrams were used to analyze the co-expression patterns of the DEGs between TG and Sp5C (Figures 2G–L). The analysis showed that 306 upregulated mRNAs and 501 downregulated mRNAs, nine upregulated and four downregulated lncRNAs, and 202 upregulated and 123 downregulated circRNAs were co-expressed in both regions. This suggests that these genes may play a significant role in response to IoN-CCI in both the TG and Sp5C. The top five up- and downregulated mRNA, lncRNA, and circRNA are shown in Supplementary Figures S3–S5. The detailed co-expressed DE mRNAs, DE lncRNAs, and DE circRNAs are listed in Supplementary Table S2.

**FIGURE 3:**
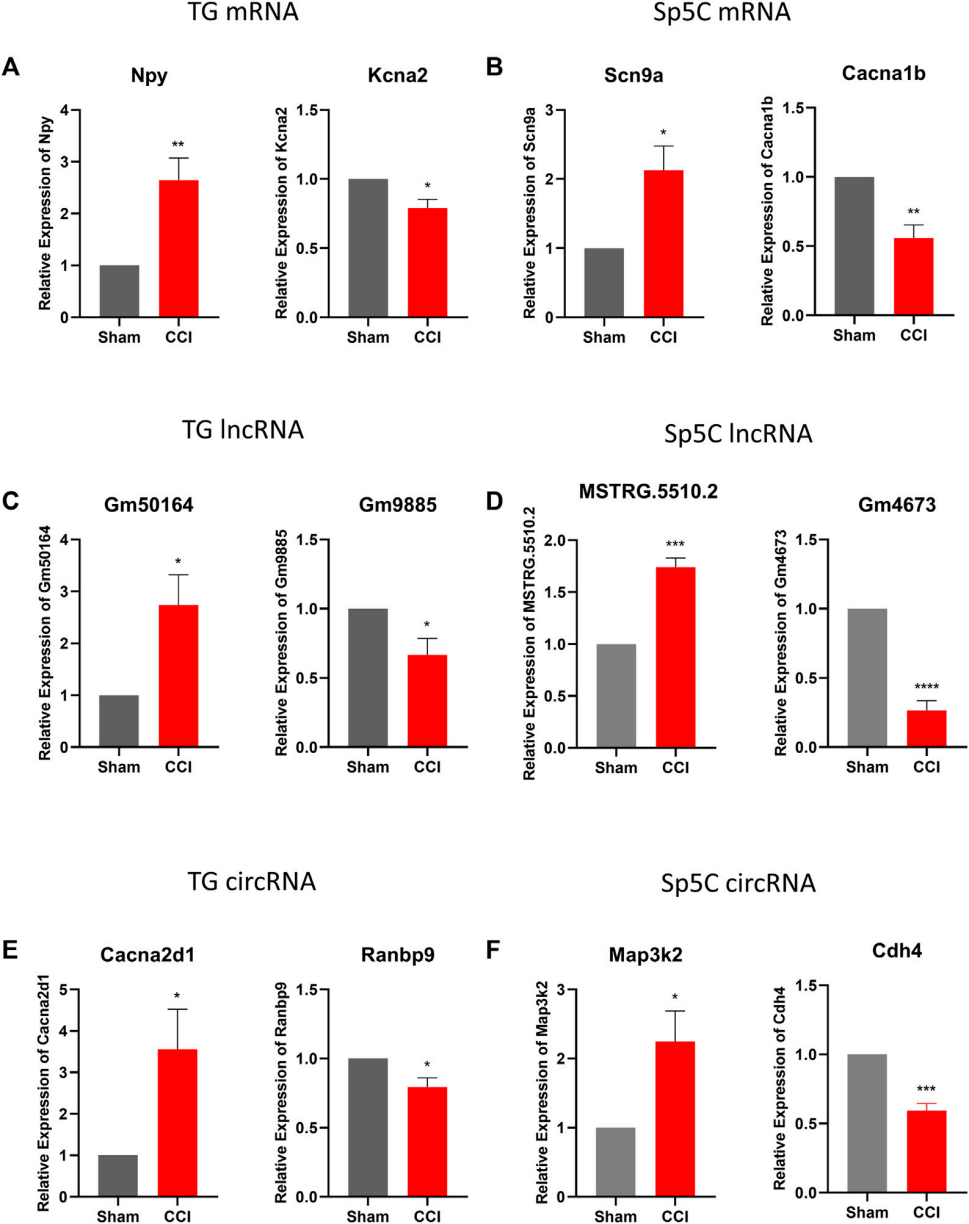
Validations of differentially expressed mRNA, lncRNAs, and circRNAs in TG and Sp5C after IoN-CCI. **(A, C, E)** Levels of mRNAs Npy and Kcna2, lncRNAs Gm50164 and Gm9885, and circRNAs Cacna2dl and Ranbp9 in TG on day 7 after IoN-CCI. n = 6 mice/group. *p < 0.05; **p < 0.01 *versus* the corresponding sham group by two-tailed unpaired Student’s t-test. **(B, D, F)** Amounts of mRNAs Scn9a and Cacan1b, lncRNAs MSTRG.5510.2 and Gm4673, and circRNAs Map3k2 and cdh4 in Sp5c on day 7 after IoN-CCI. n = 12 mice/group. *p < 0.05; **p < 0.01, *versus* the corresponding sham group by two-tailed unpaired Student’s t-test.

To confirm the accuracy and reliability of the RNA sequencing data, we performed a quantitative real-time RT-PCR assay to assess the expression level of significant DE mRNAs, lncRNAs, and circRNAs in TG and Sp5C on day 7 following IoN-CCI in two regions (Figure 3). Specifically, we analyzed the expression of two mRNAs (Npy and Kcan2), two lncRNAs (Gm50164 and Gm9885), and two cirRNA (Cacna2d1 and Ranbp9) in TG, as well as two mRNAs (Scn9a and Cacna1b), two lncRNAs (MSTRG.5510.2 and Gm4673), and two cirRNA (Map3k2 and Cdh4) in Sp5C. Our results revealed that the expression levels of these selected RNAs were consistent with the sequencing results.

### 3.4 G protein-coupled receptor and ion channel mRNAs with the highest differential expression in the TG and Sp5C after IoN-CCI

**FIGURE 4:**
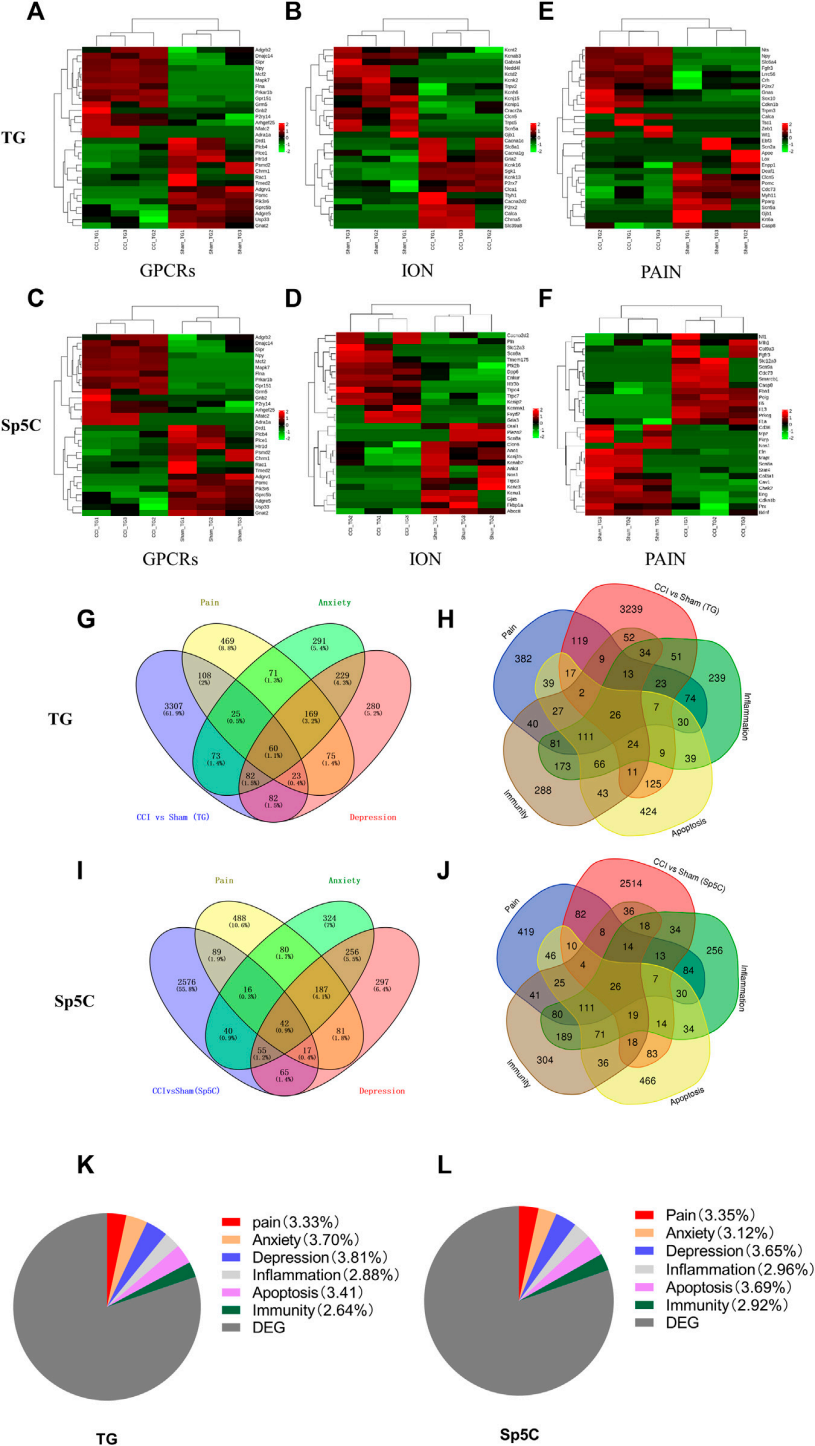
Heatmaps of the representative differentially expressed genes (DEGs) in the TG and Sp5C after nerve injury. **(A–C)** Top 15 up- and downregulated genes of G protein-coupled receptors **(A)**, ion channels **(B)**, and pain **(C)** in the TG on day 7 after CCI-ION. **(D–F)** Top 15 up- and downregulated genes of G protein-coupled receptors **(D)**, ion channels **(E)**, and pain **(F)** in the Sp5C on day 7 after CCI-ION. Colors in the heatmaps indicate the Row Z-Score among the different datasets. The up- and downregulated genes are colored in red and green, respectively. n = 9 mice/group. Venn diagrams indicate the number of DEGs mapped to pain, depression, and anxiety in the TG **(G)** and Sp5C **(I)** and the number of DEGs mapped to pain-, apoptosis-, inflammation-, and immunity-related genes in the TG **(H)** and Sp5C **(J)**. Pie graph indicates the proportions of pain-, anxiety-, depression-, inflammation-, apoptosis-, and immunity-related genes in the TG **(K)** and Sp5C DEGs **(L)**.

Ion channels and G protein-coupled receptors (GPCRs) play critical roles in the transmission and modulation of nociceptive information (Zhao et al., 2013; Campbell and Smrcka, 2018; Kumari et al., 2022). Following IoN-CCI, approximately 116 GPCR mRNAs (56 upregulated and 60 downregulated) and 84 GPCR mRNAs (43 upregulated and 41 downregulated) were identified in TG and Sp5C, respectively. Furthermore, approximately 135 ion channel mRNAs (45 upregulated and 90 downregulated) and 70 ion channel mRNAs (32 upregulated and 38 downregulated) were identified in TG and Sp5C, respectively. The top 15 GPCR and ion channel DEGs that are up- and downregulated throughout the two regions are shown in the heatmaps (Figures 4A–D). Consistent with earlier findings of pain models (Magnussen et al., 2015; Holmes et al., 2017), the levels of GPCRs such as Npy, Adra1a, Gpr151, Sstr2, P2ry12, and Adrb3 were significantly increased during IoN-CCI. In contrast, after IoN-CCI, the levels of GPCR such as Chrm1 and Htr1d decreased dramatically. We found that after IoN-CCI, the expression of the ion channels Cacna1c, Cacna2d2, P2rx2, and P2rx7 was considerably increased while Gabra4 was reported as downregulated (LaCroix-Fralish et al., 2007; Perkins et al., 2013; Reinhold et al., 2015; Liu et al., 2021).

### 3.5 Associations between differentially expressed mRNAs and pain-related emotional disorders in TG and Sp5C following IoN-CCI

In order to obtain more information regarding gene adaptations in response to IoN-CCI, we compared pain-related DEGs in the TG and Sp5C. The heatmaps of the top 15 representative up- and downregulated pain-related DEGs in the TG and Sp5C are shown in Figures 4E,F, respectively. Approximately 113 pain-related mRNAs (42 upregulated and 71 downregulated) and 74 pain-related mRNAs (32 upregulated and 42 downregulated) were identified in TG and Sp5C, respectively. For the pain-related gene, we observed the noticeably elevated expression of Slc6a4, Npy, Fgfr3, Zeb1, Cdkn1b, Nts, P2rx7, Calca, Lrrc56, Sox10, Tsc1, Wt1, Crh, Trpm3, and Gnas in the TG while Scn5a, Deaf1, Casp8, Clcn5, Pparg, Pomc, Ebf3, Myh11, Lox, Cdc73, Apoe, Gjb1, Enpp1, Scn2a, Krt6a, and Kcna2 were significantly reduced in the TG after IoN-CCI (Figure 3E). In the case of Sp5C, Scn9a, Col9a3, Polg, Casp8, Mlh1, Cdc73, Fgfr3, Nf1, Il13, Smarcb1, Prkcg, Il1a, Fbn1, Slc12a3, and Il6 were remarkably increased. In contrast, the amounts of pain-related genes in Sp5C such as Scn8a, Mpz, Chek2, Eln, Prx, Bdnf, Nos1, Fkrp, Mapt, Cd36, Stat4, Cav1, Cdkn1b, Eng, and Col3a1 were dramatically decreased after IoN-CCI (Figure 3F).

The DEGs associated with pain and emotional disorders (such as anxiety and depression) in the TG and Sp5C were then characterized using the GeneCards databases. We compared them to 3934 DEGs on TG and 5,205 on Sp5C associated with pain and emotional disorders. According to the relevance score, the TG had 85 pain- and anxiety-related DEGs, 83 depression-related DEGs, and 60 anxiety- and depression-related DEGs (Figure 4G). Sp5C had 58 pain- and anxiety-related DEGs, 59 depression-related DEGs, and 42 pain-, anxiety-, and depression-related DEGs (Figure 4I). Both TG and Sp5C had 30 pain- and anxiety-related DEGs, 29 pain- and depression-related DEGs, and 23 pain-, anxiety-, and depression-related DEGs. Several shared DEGs are associated with pain, depression, and anxiety in the TG and Sp5C. TG shares 60 DEGs (1.1%) and Sp5C 42 DEGs (0.9%) between pain, depression, and anxiety (Supplementary Table S3).

The DEGs involved in neuroinflammation, apoptosis, immunity, and pain were presented via a Venn diagram. Pain-, apoptosis-, inflammation-, and immunity-related genes in the TG and Sp5C share a total of 26 DEGs. Based on the relevance score, the TG had 52 pain- and apoptosis-related DEGs, 69 pain- and inflammation-related DEGs, 50 pain- and immunity-related DEGs, and 26 pain-, apoptosis-, inflammation-, and immunity-related DEGs (Figure 4H). Approximately 47 DEGs associated with pain and apoptosis, 60 with pain and inflammation, 52 with pain and immunity, and 26 with pain, apoptosis, inflammation, and immunity were found in the Sp5C (Figure 4J). Both TG and Sp5C had seven pain-, apoptosis-, inflammation-, and immunity-related DEGs (Supplementary Table S4). The proportions of pain-, anxiety-, depression-, neuroinflammation-, apoptosis-, and immunity-related genes in the TG (Figure 4K) were 3.33%, 3.70%, 3.81%, 2.88%, 3.41%, and 2.64%, respectively, and the proportions of them in the Sp5C (Figure 4L) were 3.35%, 3.12%, 3.65%, 2.96%, 3.69%, and 2.92%, respectively.

### 3.6 DE ncRNA-targeting genes predominate in nociception-related signaling pathways

**FIGURE 5:**
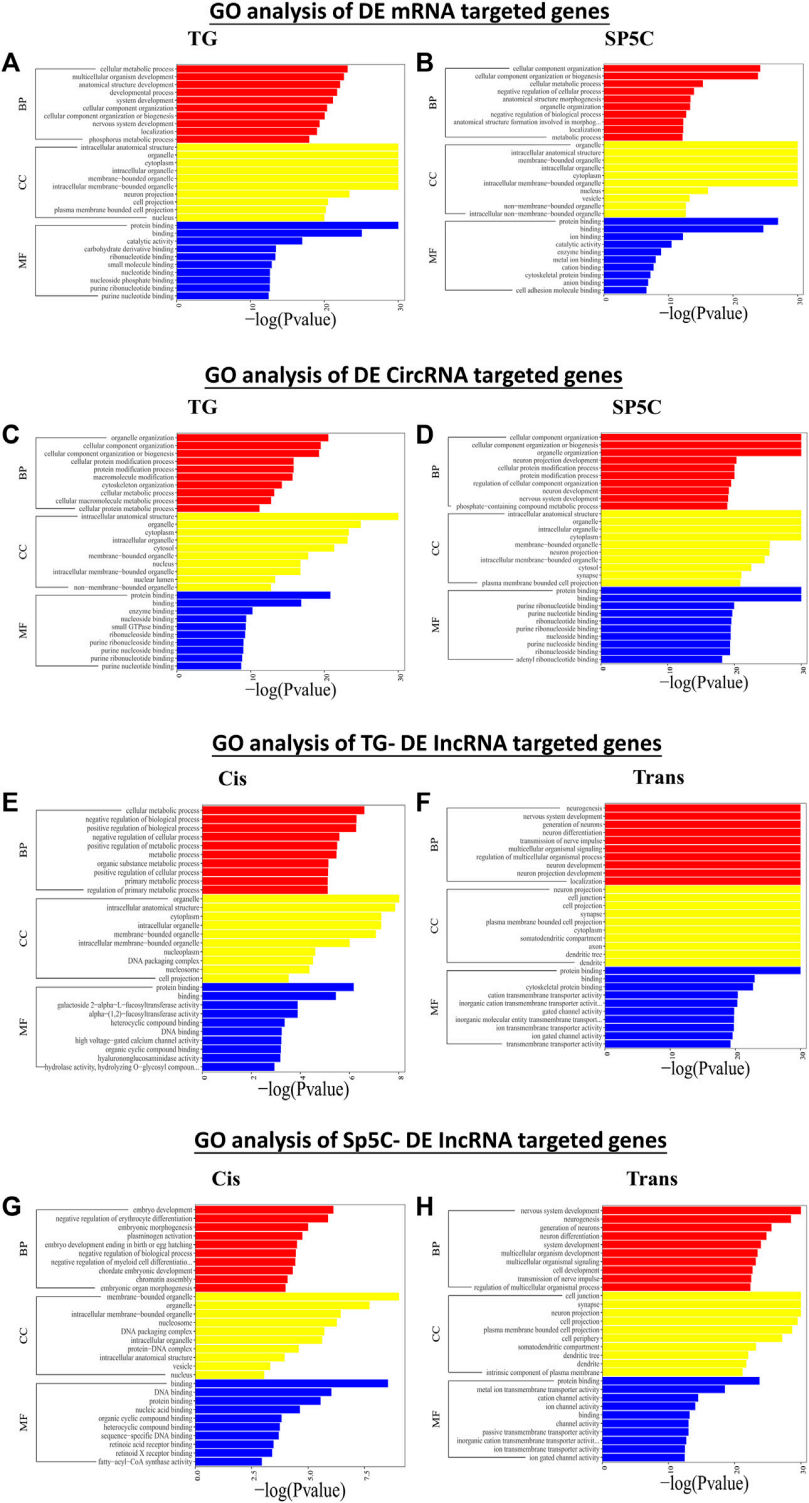
Functional prediction of DE mRNAs in TG **(A)** and SP5C **(B)**, DE circRNAs in TG **(C)** and SP5C **(D)**, DE cis-lncRNAs **(E)** and cis-lncRNAs **(F)** in TG, and DE cis-lncRNAs **(G)** and cis-lncRNAs **(H)** in SP5C of CCI mice by GO analyses. Analysis of the Gene Ontology database graphically displays the top 10 significant GO enrichment results with the candidate targeted genes in the biological process, molecular function, and cellular component in the TG and SP5C. Significantly enriched GO bar chart, screening significantly enriched GO according to the *p*-value less than or equal to 0.05, with up to 10 GO categories, the abscissa represents -log10 (*p*-value), and the ordinate represents the significantly enriched GO name.

According to the GO analysis of target genes of DE mRNAs across two regions, significant biological processes (BPs) were cellular metabolic process, nervous system development, biogenesis, and localization, while organelle, neuron, and cell projections were the noteworthy cellular component (CC) enrichments (Figures 5A,B). The most robust molecular functions (MFs) in both TG and Sp5C were enriched in binding, catalytic activity, and carbohydrate derivate binding. The co-localization and co-expression genes of circRNAs and the DE lncRNA analysis display somehow similar enrichments of BP, MF, and CC (Figures 5A–H). The DE trans-lncRNA’s most significant BP enrichments were neurogenesis, nervous system development, neuron differentiation, generation of neurons, and transmission of nerve impulse, and the noteworthy CC enrichments were seen in neuron projection, cell junction, cell projection, synapse, axon, and dendrite.

**FIGURE 6:**
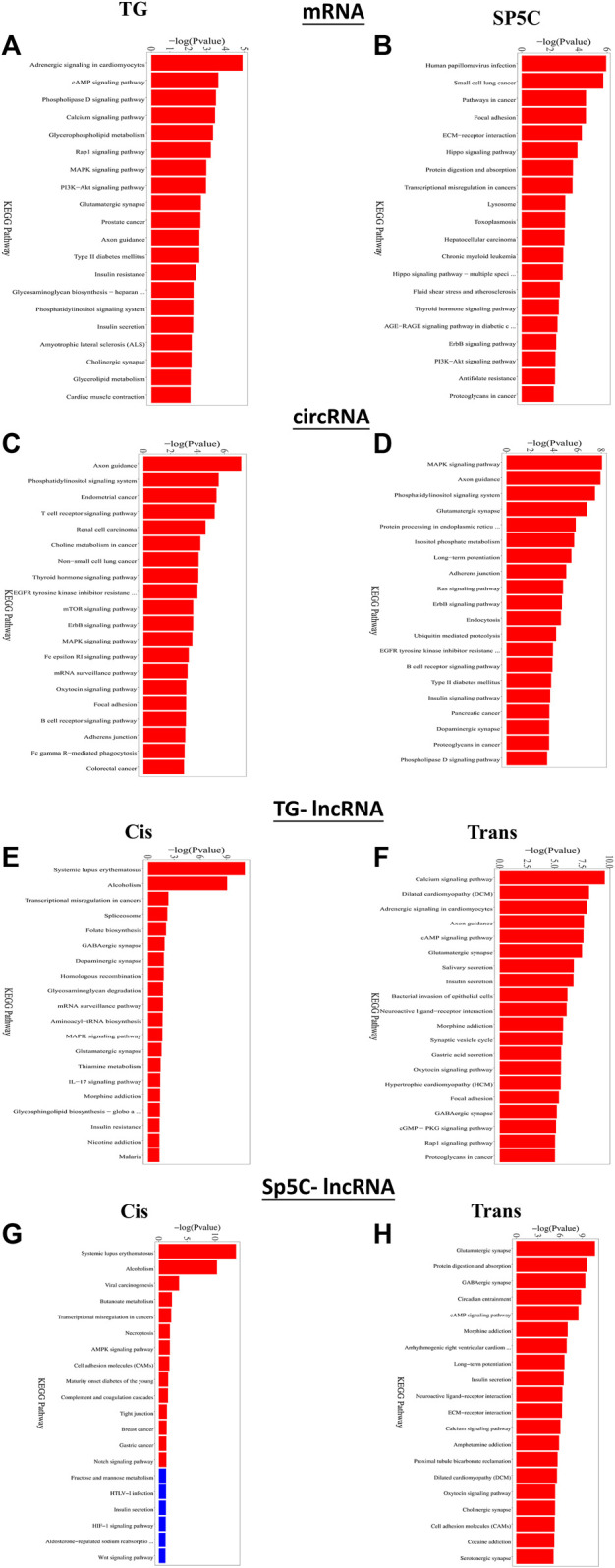
Functional prediction of DE mRNAs in the TG **(A)** and SP5C **(B)**, DE circRNAs in TG **(C)** and SP5C **(D)**, DE cis-lncRNAs **(E)** and cis-lncRNAs **(F)** in TG and DE cis-lncRNAs **(G)** and cis-lncRNAs **(H)** in SP5C of CCI mice by KEGG analyses showed top 10 enrichments of KEGG pathways of CCI mice. A significantly enriched KEGG pathway bar graph is shown; the abscissa represents the significantly enriched KEGG pathway name, and the ordinate represents -log10 (*p*-value). The more significant the ordinate is, the more significant the pathway enrichment, and the red column indicates the significant pathway (*p*-value <= 0.05).

Furthermore, KEGG analysis of predicted genes of DE mRNAs showed the most significantly enriched pathways were adrenergic signaling, cAMP signaling, MAPK signaling, PI3K-Akt signaling, and Rap1 signaling (Figures 6A,B). Predicted genes of DE circRNAs of IoN-CCI mice were significantly enriched with axon guidance, the T-cell receptor signaling pathway, the mTOR signaling pathway, and the MAPK signaling pathway (Figures 6C,D). KEGG analysis of anticipated genes of DE cis- and trans-lncRNAs showed GABAergic, MAPK, RAP1, adrenergic, and cAMP signaling pathways (Figures 6E–H). The up- and downregulated pain-related genes across both regions of IoN-CCI, their functions, and pathways involved are listed in Supplementary Tables S5–S6. Both regions of IoN-CCI mice had KEGG analysis pathways mostly associated with hyperalgesia and pain nociception (Ji et al., 2009; Eijkelkamp et al., 2010; Wang et al., 2021).

The top 50 DEGs with the highest correlation degree were filtered out of the STRING database to construct a network of DEGs related to pain in TG and Sp5C (Supplementary Figure S6). The DEGs, such as Nfkb1, Ctnnb1, RAC1, Ephb2, Irak1, Irak2, Jak1, Map3k7 (also known as TAK1), Mapk1 (also known as Erk2), Mapk14 (also known as p38), Akt1, Akt2, Fgfr1, Fgfr3, Lepr, and Ank3 were found as important molecules among the hub genes across the TG. The important molecules from the hub genes of the Sp5C included Casp3, Nfkb1, Ank3, Egfr, Frk, Epha5, Nos1, Fgfr3, Abl1, Il6, Ctnnb1, Igf1r, Epha1, Axl, Hck, Lrrc47, Actr1b, Tie1, Pik3c3, Stk11, Trp53, and Zap70. Most of the hub genes and their partner genes across the TG and Sp5C are involved in neuropeptide signaling pathways (LaCroix-Fralish et al., 2007).

### 3.7 CeRNA network analysis

**FIGURE 7:**
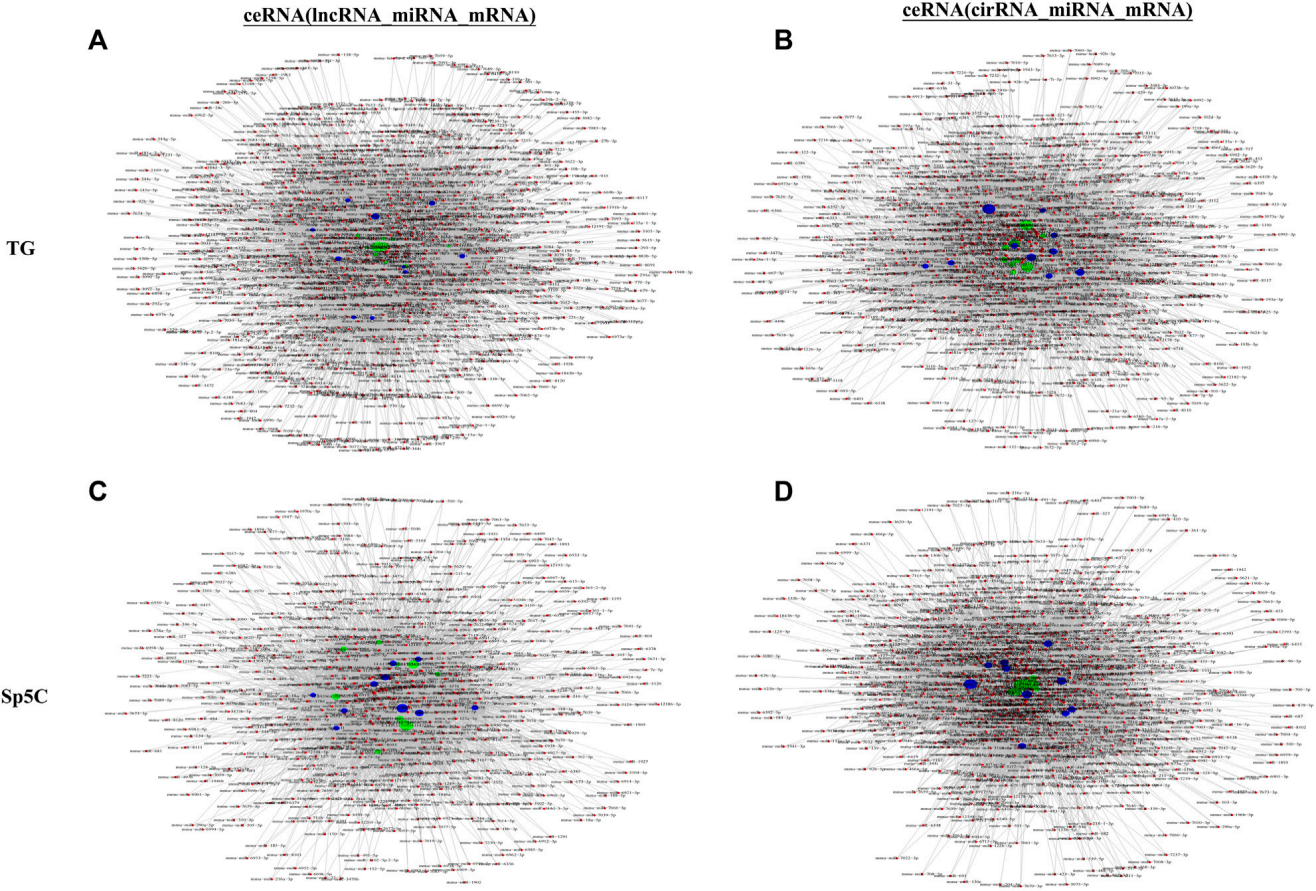
Potential interaction between miRNA–long non-coding RNA (lncRNA)–mRNA networks and miRNA–circular RNA (cirRNA)–mRNA networks in TG regions after CCI-IoN. The ceRNA networks were constructed based on correlation analysis. Red nodes represent miRNAs, green nodes represent lncRNAs, purple nodes represent cirRNAs, and blue nodes represent mRNAs. The ceRNA regulatory network was constructed with the same direction of differential expression (generally positive correlation); otherwise, the ceRNA regulatory network was constructed between multiple groups or repeated samples based on both *p*-value ≤ 0.05 and PPC ≥ 0.5 (generally positive correlation). The top 10 lncRNAs and cirRNAs and their target genes are displayed according to their respective network degrees of mRNA, lncRNAs, and cirRNAs. **(A, C)** ceRNA (miRNA–lncRNA–mRNA) network on TG **(A)** and Sp5C **(C)**. **(B, D)** ceRNA (miRNA–cirRNA–mRNA) network on TG **(B)** and Sp5C **(D)**.

lncRNAs and circRNAs sponge miRNAs to regulate mRNA expression, which is linked to NP. CeRNA networks were constructed in the TG and Sp5C regions using correlation analysis to investigate the potential interaction between miRNA–lncRNA–mRNA and miRNA–circRNA–mRNA networks involved in TNP. Based on the network degrees of mRNA, lncRNAs, and circRNAs, the top 10 lncRNAs (Figures 7A,C) and circRNAs (Figures 7B,D) were displayed, along with their target genes (Supplementary Tables S7–S10).

## 4 Discussion

TNP, orofacial pain caused by cranial nerve lesions or cranial nerves diseases, is a serious public health issue that typically include mechanical allodynia and hyperalgesia and lasts for over 4 weeks, even after the initial cutaneous, mucosal, or muscular damage has healed (Finnerup et al., 2021; Xu et al., 2022). TNP is usually difficult to treat by analgesics or surgical intervention (Murugappan et al., 2021). Despite substantial research into TNP’s causes, effective treatments remain elusive due to a lack of understanding (Jones et al., 2018). Evidence suggests that NP development and maintenance are significantly influenced by changes in gene expression profiles at various levels of the nervous system. Despite growing evidence that ncRNAs are crucial to NP, little is known about TG ncRNA expression and function in TNP (Zhang et al., 2019; Song et al., 2020; Fang et al., 2022). In recent years, it has come to light that a number of lncRNAs are responsible for regulating various form of pain (Zhao et al., 2013; Dong et al., 2014; Cai et al., 2020; Kalpachidou et al., 2020; Song et al., 2020; Xu et al., 2020; Pan et al., 2021; Liu et al., 2022). However, the potential roles that lncRNAs may play in orofacial pain remain unclear. To the best of our knowledge, this study is the first to use whole-transcriptome sequencing and bioinformatics analysis to look at the expression profile of ncRNAs in the TG and Sp5C of IoN-CCI mice.

NP is characterized by both sensory and affective disturbances, and this frequent comorbidity supports the notion that pain and mood disorders share some common pathogenetic mechanisms. The affective disturbances associated with pain include working memory dysfunction, impaired cognition, decreased appetite, depression, anhedonia, disruptions to sleep cycles, and impaired familial and social interactions (Aloisi et al., 2016). The study revealed several DEGs associated with pain or emotional dysfunction, including GPCRs and ion channels. Npy, one of the upregulated GPCRs in the DEGs, was found to be elevated in the dorsal root ganglia and spinal cord, indicating a compensatory response to nerve damage (Basu et al., 2022; Chalaki et al., 2022). Additionally, the GPR151 receptor in nociceptors was identified to regulate P2X3 function and microglial activation, thereby modulating NP (Xia et al., 2021). Similarly, we observed changes in the expression of ion channels following IoN-CCI. Among the upregulated ion channel, the increased expression of Cav1.2 channels in sensory neurons can lead to enhanced calcium influx and hyperexcitability, which can result in pain hypersensitivity (Jeon et al., 2010; Radwani et al., 2016). Conversely, reducing the activity of these channels through genetic or pharmacological means can attenuate NP in animal models. Moreover, blocking or reducing the activity of P2X2 has been shown to pain alleviation in the spinal cord (Jarvis et al., 2002). Consistent with our sequence data, the downregulation of Gabra4 results in increased excitability and hyperalgesia (Bonin et al., 2011).

In order to predict the gene function of DE ncRNAs in the condition of TNP, GO and KEGG pathway analyses were performed. Histograms using the -log (*p*-value) of the GO terms showed the significance of enriched BP, CC, and MF. Consistent with previous reports, GO term and KEGG pathway enrichment analyses in two regions showed notable enrichments in axon guidance, the T-cell receptor signaling pathway, mTOR signaling pathway, the MAPK signaling pathway, and so on, which was similar to the results of pathway enrichment of DEGs in the spinal cord of rats treated with spared nerve injury (Zhou et al., 2017). Consistently, functional analyses revealed that a large proportion of pain-, anxiety-, and depression-related DEGs were strongly associated with neuroinflammation and apoptosis, which were believed to play a significant role in pain states (Chelyshev et al., 2001; Ji et al., 2018; Matsuda et al., 2019). Using the STRING database, we constructed a protein–protein interaction (PPI) network to better understand the potential role and analyze the functional connections of pain-related DEGs in TNP. PPI analysis consistent with previous reports (Perkins et al., 2013; Jamieson et al., 2014) indicated that several upregulated hub genes (Nfkb1 Ctnnb1, Akt2, RAC1 and Ephb2, Irak2, Irak1, Casp3, Jak1, Map3k7, and Mapk1) were involved in the pain modulation. These genes were related to nerve injury and regeneration and nociception regulation (Nfkb1, Akt2, RAC1, and Ephb2) (Liu et al., 2020; Bai et al., 2023), pro-nociceptive signaling transmission (Nos1, Jak1, and Map3k7) (Escolano-Lozano et al., 2021; Peirs et al., 2021), and anti-nociceptive signaling transmission (Akt1 and Fgfr3). In addition, it remains to be determined whether the aforementioned alterations in DEGs can serve as new therapeutic targets, despite the fact that the current study demonstrated the alterations in gene transcript and revealed their functional analyses in the TG and Sp5C. Moreover, the identification of novel genes and pathways that have not been previously documented in the literature for TNP could potentially reveal unexplored aspects of the condition. Additional investigation into unexplored molecular domains may yield valuable insights into the probable regulatory mechanisms underlying TNP, hence providing opportunities for the development of novel therapeutic strategies.

## 5 Conclusion

In conclusion, this study revealed the first TG and Sp5C gene expression profiles of mRNAs, circRNAs, and lncRNAs in the IoN-CCI pain model. This study provides findings that ncRNAs may be crucial players in the emergence of TNP, influencing both mRNAs and pain-related genes. The detailed roles of the novel DE ncRNAs and DEGs currently unrelated to pain in the TG and Sp5C of IoN-CCI mice also deserve to be investigated in the future. As a result, the findings of this research offer a fresh understanding of the mechanisms of TNP as well as potential treatment targets for the condition.

## Data Availability

The datasets presented in this study can be found in online repositories. The names of the repository/repositories and accession number(s) can be found at: https://www.ncbi.nlm.nih.gov/, SUB13606115.
