# Supplementary material for: Transcriptome-wide analysis of trigeminal ganglion and subnucleus caudalis in a mouse model of chronic constriction injury-induced trigeminal neuralgia
Source: Front Pharmacol. 2023 Sep 28;14:1230633. doi: 10.3389/fphar.2023.1230633 (PMC10568182; doi:10.3389/fphar.2023.1230633)
Supplement: Supplementary file 1 [file DataSheet1.zip › Supplementary Material/Supplementary Figures + Tables 1-6.DOCX]

Supplementary Material

Transcriptome-wide Analysis of Trigeminal Ganglion and Subnucleus Caudalis in a Mouse Model of Chronic Constriction Injury-induced Trigeminal Neuralgia

Xiaona Cui^1†^, Bo Qin^2†^, Chaoyun Xia^1,3^, Hong Li^1,3^, Zhiye Li^4^, Zhisong Li^3^, Abdul Nasir^1,3*^, Qian Bai^1,3*^

^1^Medical Research Center, The Second Affiliated Hospital of Zhengzhou University, Zhengzhou, Henan, China

^2^Translational Medical Center, The First Affiliated Hospital of Zhengzhou University, Zhengzhou, Henan, China

^3^Department of Anesthesiology and Perioperative Medicine, The Second Affiliated Hospital of Zhengzhou University, Zhengzhou, Henan, China

^4^Department of Pharmacy, the Second Affiliated Hospital of Zhengzhou University, Zhengzhou, Henan, China

^†^ These authors contributed equally.

*** Correspondence:**

Abdul Nasir, PhD, [anasir@zzu.edu.cn](mailto:anasir@zzu.edu.cn);

Qian Bai, PhD, [baiqian@zzu.edu.cn](mailto:baiqian@zzu.edu.cn)

**Figure S1:** The proportion and expression of exonic, intronic, and intergenic in sham and ION-CCI groups from TG and Sp5C, and the transcriptional changes of mRNAs and lncRNAs in TG and Sp5C after ION-CCI. (A)The total proportion of the mapped reads of each category in sham and CCI groups in TG and Sp5C areas. (B, C) Changes of exonic, intronic and intergenic expression upon ION-CCI procedure in TG and Sp5C. n = 9 mice/group. *P < 0.05; **P < 0.01, and ***P < 0.001 versus the corresponding sham group by two-tailed unpaired Student s t-test. (D, E) The proportion of protein-coding RNAs, other non-coding RNAs, know lncRNAs and predicted lncRNAs. (F)The densities of transcript length and exons numbers of protein-coding RNAs, know lncRNAs and predicted lncRNAs.

**Figure S2:** The changes in gene expression of mRNAs (A-B), lncRNAs (C-D) and circRNAs (E-F) within the TG and Sp5C after ION-CCI.

**Figure S3:** PPI network establishments to analyze protein–protein interactions. The top 50 differentially expressed genes (DEGs) were picked out based on the connection degree of genes and constructed the network in the mRNAs (A-B), lncRNAs (C-D) and circRNAs (E-F) within the TG and Sp5C after ION-CCI.

**Table legends**

**Table S1**: Primers for RT-qPCR

**Table S2**: List of up- and downregulated mRNA, lncRNA and cirRNA in TG and Sp5C

**Table S3**: Overlapped genes between pain and Anxiety/Depression related genes.

**Table S4**: Overlapped genes between pain and Inflammation/Apoptosis/Immunity related genes.

**Table S5**: Upregulated pain-related genes, their function and pathway involved.

**Table S6**: Downregulated pain-related genes, their function and pathway involved.

**Table S7**: ceRNA network of Top 10 lncRNA_miRNA_mRNA in TG

**Table S8**: ceRNA network of Top 10 lncRNA_miRNA_mRNA in Sp5C

**Table S9**: ceRNA network of Top 10 cirRNA_miRNA_mRNA in TG

**Table S10**: ceRNA network of Top 10 cirRNA _miRNA_mRNA in Sp5C


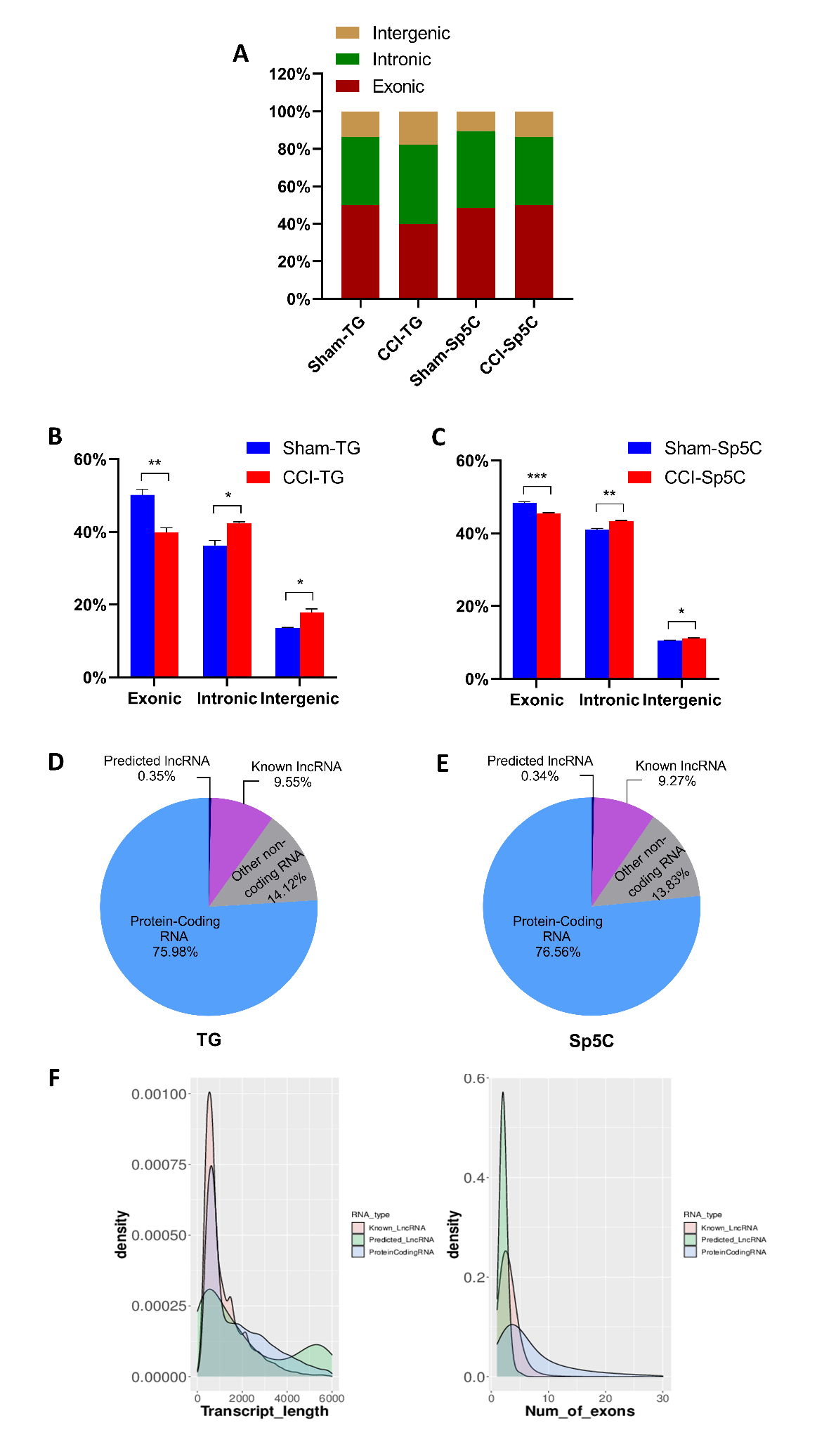

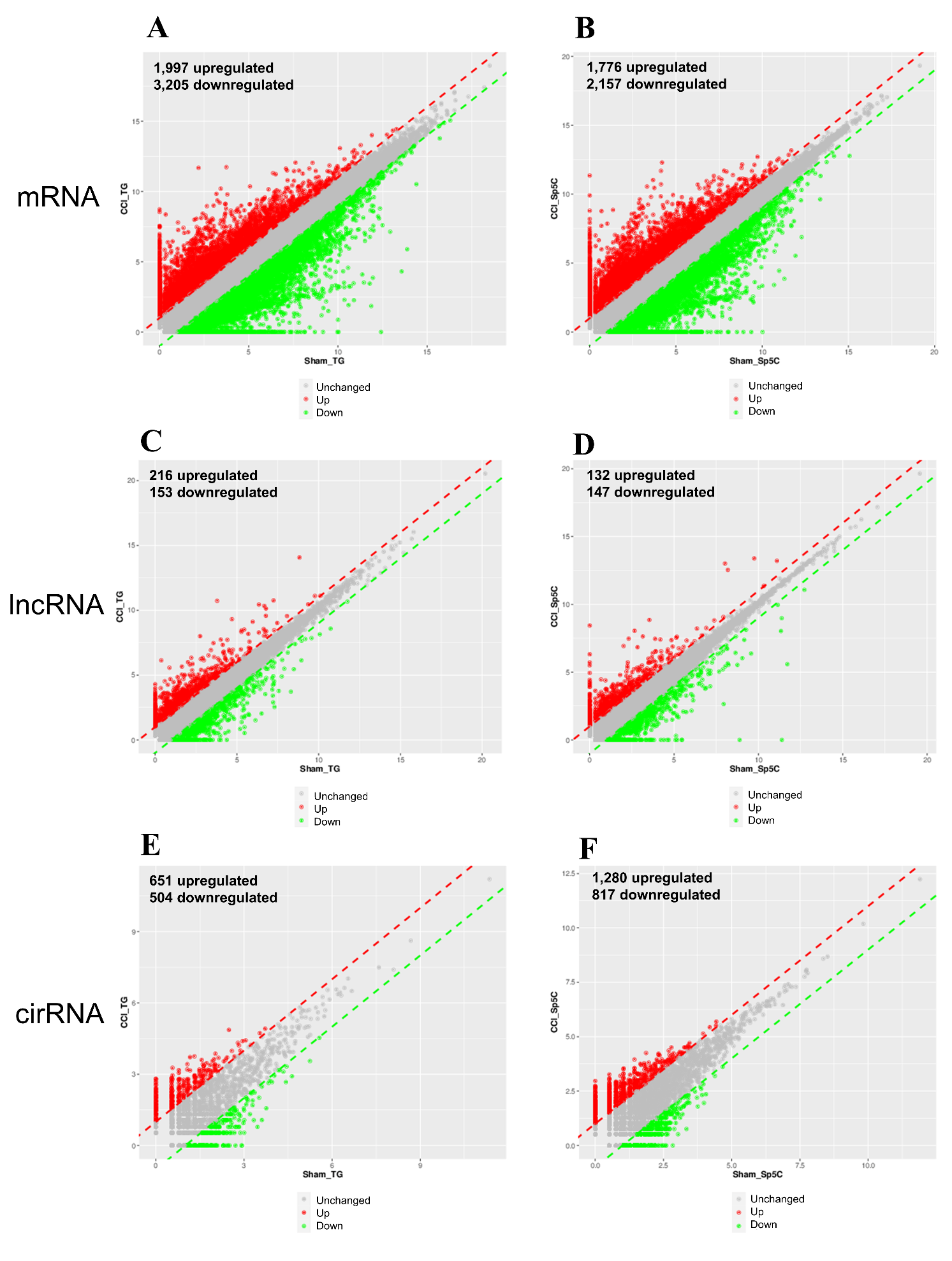

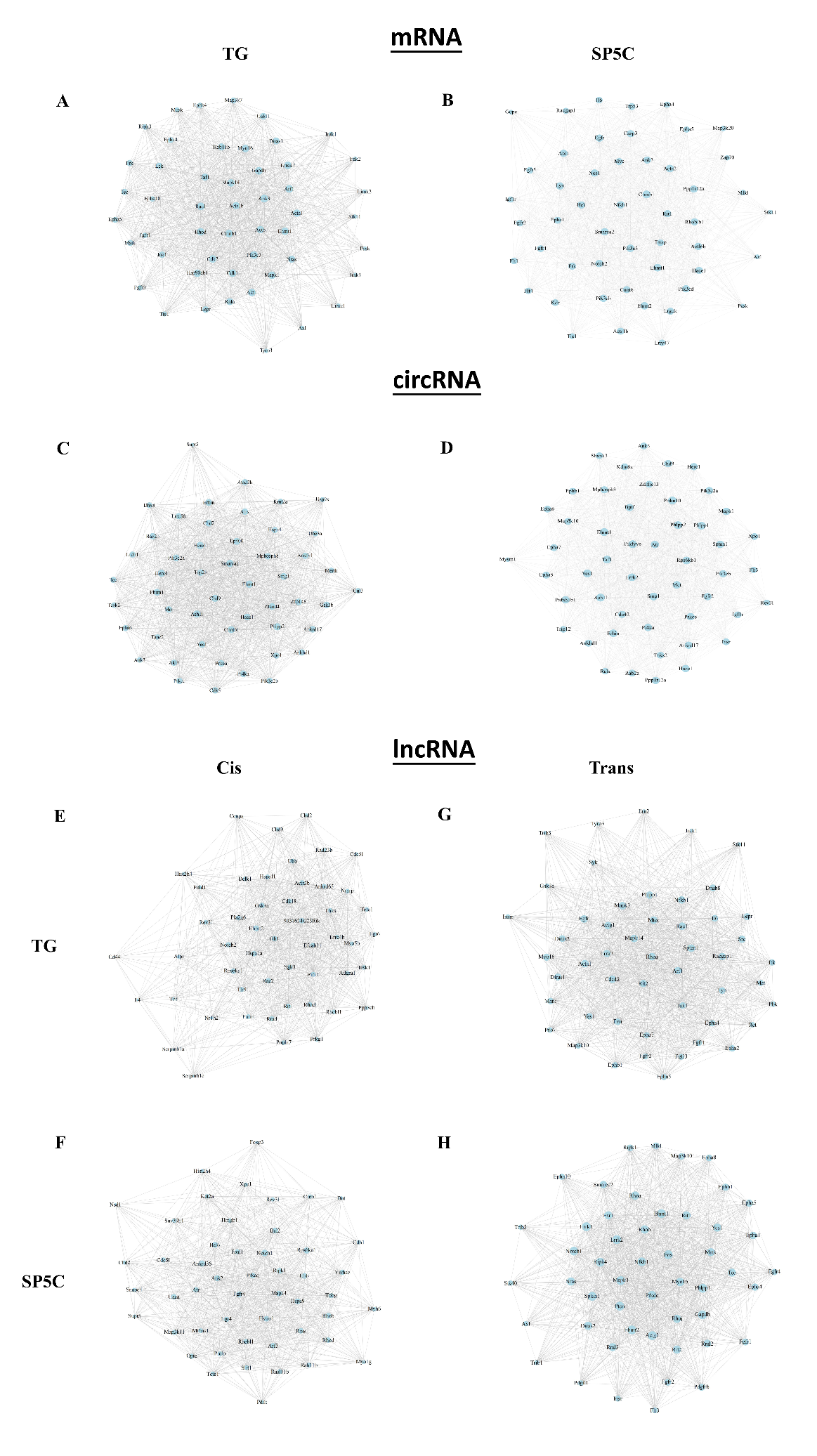


**Table S1.** Primers for RT-qPCR

| **Primer names** | **Sequences** |
| --- | --- |
| Npy-S | TCCGCTCTGCGACACTACATC |
| Npy-A | AAGGGTCTTCAAGCCTTGTTCT |
| Kcna2-S | GGTGTGGCTGCTCTTTGAGTATC |
| Kcna2-A | CCGTGAAGTCCTTGTCGTCCT |
| Scn9a-S | TGGACTACAACTGTTTATGGGCAA |
| Scn9a-A | TGAGTCATTAGCCGAAACAAGG |
| Cacna1b-S | CATCGTGGTCTTCGCTCTGTT |
| Cacna1b-A | CACCTTGCGACTCAATCCCAT |
| Gm50164-S | GGGAGAAACTTGAGAAGTAGAGCCT |
| Gm50164-A | CCTTCATGTTGTCTGTTGGGTG |
| Gm9885-S | CGAGGCAAGTTTAGACCTGATG |
| Gm9885-A | CTTTCAGATCCTGGATGCTGTTG |
| MSTRG.5510.2-S | CCTCTACCATCATCATGAGATATGC |
| MSTRG.5510.2-A | GCGAAAGGTAAACGGAGGAAT |
| Gm4673-S | GGGCACAGGGTTTGAAGACT |
| Gm4673-A | CCCCACTATTATGATGATTTGAGG |
| Cacna2d1-S | TTCAGAACTCTGGTCAAGTCTCAAG |
| Cacna2d1-A | ACGGCATCTTTCAACACCTTCT |
| Ranbp9-S | ACAGTTATCCCGTCAGTCCTCG |
| Ranbp9-A | TGGCATCAACCACTTCTCCTG |
| Map3k2-S | ACGAGGAAGTACACCCGTCAGA |
| Map3k2-A | AAGATAATGCTGGCCGACTGG |
| Cdh4-S | ATGAACGGTAACAAGGTGGAGAA |
| Cdh4-A | ATACATCCGGCCAGACATAGAGT |
| GAPDH-S | CCTCGTCCCGTAGACAAAATG |
| GAPDH-A | TGAGGTCAATGAAGGGGTCGT |

RT-qPCR, reversed transcriptase quantitative polymerase chain reaction; S, sense; A, anti-sense

**Table S2.** List of up- and downregulated mRNA, lncRNA and cirRNA in TG and Sp5C

|  | **TG+ and Sp5C+** | **TG- and Sp5C-** |
| --- | --- | --- |
| **mRNA** | **306 genes**  Per1,Anxa10,Vangl2,0610010F05Rik,Zmym5,Nin,Rbm6,Mapk7,Dpy19l4,Ctage5,Armc2,Numb,Mink1,Elmo3,Rnasel,Cacna1c,Mto1,Tardbp,Prss23,Grm5,Lrig1,Ccm2,Fgfr3,Tsc22d1,Pcdh15,Gpatch4,Mpped1,Wdr53,Fam105a,Ddr1,Rgs12,Gpm6b,Arhgef25,Myo6,Apbb2,Cacna2d2,P2ry14,Smc6,Anp32e,Atf3,Gnb2,Tnr,Fbxl5,Rasgrp2,Smarcad1,Sorbs2,Cd2bp2,Homer3,Psma6,Fam228b,Szrd1,Telo2,Zmynd11,Glrx,Gigyf2,Pik3c3,Tmem161b,Lrsam1,Cenpo,Adgrl2,Tdrd7,Cadps2,Abhd14a,Map4k5,Pik3cd,Ttyh1,Zmym3,Mob1a,Atp13a5,Elk3,Tuba4a,Ywhag,Papd5,Rims1,Afdn,Clpb,Vps53,Grip1,4930402H24Rik,Mknk1,Efemp2,2310035C23Rik,Zfp2,Poli,Vps29,Gm44790,Clec5a,Sspo,Stk38,Pxdc1,Slc9a8,0610037L13Rik,Ablim2,Gnas,Dusp14,Cep192,Plch1,Art3,Naa60,Kif1bp,Nipsnap1,Usp28,Ahcyl2,Gcgm,Prdx6,Fmr1,Mtf2,Hmgn3,Grik1,Nxf1,Mum1l1,Hps3,Luzp1,Slc12a3,Etl4,Zfp934,Psmd8,Pigx,Malt1,Phf21a,U2af1l4,Tmem87a,Tmem41b,Slc29a1,Trim14,Gria3,Ninl,Tnik,Map9,Slc26a6,Cdca2,Pbrm1,Milr1,Zfp148,Foxk1,Nkiras1,Hyal2,Tom1l1,Ly6a,Phf6,Clvs1,Ehmt1,Smco3, 8-Mar,Rbm39,Adam17,Cinp,Fyttd1,Glyat,Ciz1,Xlr3a,Rb1cc1,Il15ra,Clasrp,Phkg2,Uhrf1,Phc3,Tm2d3,Trps1,Spopl,Erc2,Clec16a,Aebp2,Dhx32,Ccl12,Kcnma1,Ly6e,Abcb8,Ablim1,Lsm7,Col24a1,Mgst2,Cobll1,Ikbkg,Rrnad1,Fam229b,Arhgap45,Dtnb,Cbx3,D3Ertd751e,4931406C07Rik,Odf2,Brd4,Scmh1,Fuom,Wars2,Oprl1,Ddx41,Fsip2,Neurl4,Trim2,Nf1,Cd300c2,C2cd5,Ppp2r2d,Homez,Rpap1,Ogt,Hdac6,Sipa1l3,Ccar1,Gm13889,Ctla2a,Kctd20,Usp49,Zbtb20,Zbtb38,Mbd1,Chid1,Cacna1a,Coro7,Adamts17,Gstm6,Tenm4,Tmem164,Zcchc11,Traf3,Herc3,Nipal3,Cyb5r1,Isca2,Zbtb6,Cacna2d1,Ctss,Tor1aip2,Hddc2,Tm9sf4,Fyb,Ankrd44,Gtdc1,Foxp1,Zfp943,2010111I01Rik,Hnrnpk,Ctc1,Rars2,Zfp638,Traf3ip1,Alkbh8,Gpr155,Bag6,Parvg,Eno4,Mtus1,Ptpmt1,Ing1,Macrod2,Fbxw9,Pdss2,Tcf3,Bcas3,Mrps10,Lamb1,Fars2,Amotl1,Tcf12,Pkib,Col9a3,Ccnl1,Pde1c,Chl1,Zfx,Fbn1,1110032A03Rik,Lrrtm4,Prpf38b,Prdx1,Tacc2,Ptgr2,Bbs7,Uxt,Syt6,Atp9bMyl6,Nemf,Tmem19,Cspp1,Slc25a22,Mrps18c,Synpo2,Invs,Tead1,Enkd1,Masp2,AasdhZmiz2,Kpna1,Xaf1,Cep128,Manba,Adgrb3,Ufd1,Itsn1,Cfap69,1110017D15Rik,Cln3,Fkbp3,Swi5,Sytl2,Scml4,Manf,Atraid,Mydgf,Plec,Mff | **501 genes**  Ccser2,Sik2,Svop,Eif4a3,Dok1,Smad6,Napepld,Rab11fip3,Eif4enif1,Zfp316,Col1a2,Nrg1Map1lc3a,Tubgcp3,Atp6v0b,St3gal6,Neurl4,Fgf13Emc1,Adam23,Gatd1,Capzb,Adam15,Exoc3l,Nipal3,Pgap2,Dhx30,Tecpr2,Stxbp2,Lrrc32,Mapt,Dapk3,Col6a3,Zfp934,Arhgef4,Lgals3bp,Hs6st2,Gramd1a,Asb2,Ace,Zzz3,E2f1,Robo4,Eml1,Lcp1,Alas1,Pnkd,Fbxo30,6430548M08Rik,Synj2,Nipa2,Dlg4,Pdlim5Tmem214,Pla2g6,Adgrg1,Kif1bp,Shisa5,Ccdc88c,Ubr4,Bgn,Elf2,Cryab,Eef1d,Tie1,Rabep1,Phf24,Hdac7,Scube2,Fnbp1,Aebp1,Atxn2,Palld,Trmt1,E2f3,Dtnbp1,Ablim1,Hpca,Abhd11,Prom1,Kirrel,Med13lZfp740,Ube4b,Palm,Akap13,Pcnx3,Mat2b,Vwf,Fgfr1,Mrpl33,Mxra8,Tmtc4,Prrxl1,Ptbp1,Svil,Mesd,Arfrp1,Lrrc27,Dus1l,Ptprk,M,ml1,Ubap2l,Arfgap1,Myo1c,Tead1,H2-Ke6,2310003L06Rik,Tbc1d24,Ahcyl2,Mpp4,Men1,Nfic,Actl6b,Col1a1,Chd5,Tcf25,Syngr2,Tle2,Tnc,Taz,Kdelc1,Nsmf,Sec24c,Clip3,Atxn7l1,Frmd5,Trim23,Skiv2l,Myo5a,Aox3,Casc1,Rnf220,Camk2g,Cdkn1b,Tfcp2l1,Zyx,Ank1,Ptk2b,Cytl1,Tmem147,Arhgap29,Clcn6Frmd4b,Nt5dc2,Ptgir,Clasp1,Mtmr12,Actr1b,Tpm1,Ccpg1,Cacna2d2,Sema6c,Enpp3,Ndst2,Rab6b,Acbd4,Rhbdl2,Camk2dDlg2,Bnc2,Fbxo34,Esm1,Fmnl3,Arpp21,Osbpl1a,Nek8,Raly,Prosc,Erg,Ankrd27,Nlgn3,Ripor3,Pcmt1,Zfand4,Acot7,Cstf2,Rell2,Cox6b2,Ccl27a,Mcf2l,Serpinh1,Pnisr,Arhgef40,Morf4l2,Abr,Kcnab2,Dlg1,Trip12,Rpl22l1,Rab14,Sharpin,Rxrb,Abcb8,Celf4,Zfp618,Snapc4,Ltc4s,Tacc2,Snap91,Gnb2,Mmp23,Tab3,Cacna1b,Smtn,Serhl,Chd4,Spats2l,Ciz1,Zfp426,Tmem229b,Nup214,Hyal2,Csmd3,Il10rb,Acsl6,Usp29,Acacb,Ntrk2,B3galnt2,Stx5a,Crispld2,Mecom,Slc2a5,Gbf1,Selenos,Dab2ip,Fbxo22,Cyfip1,Crk,Phtf1,Mink1,Nisch,Grb2,Lrrc47,Hnrnpk,Slc35c2,Zfx,Trps1,Lrch4,Glb1l2,Numb,Zfp827,Cers4,Tmem234,Trak1,Egfl7,Cnot1,Cant1,Gpatch4,Mark2,Medag,Gas7,Timeless,Acvr1,Pear1,Susd2,Fam20b,Pth1r,Cadps2,Izumo4,Celf5,Rasal3,Capn11,Myrf,Prx,Mas1,Nup93,Fbxo40,Arhgap27,Emp1,Sapcd1,4930402H24Rik,Mbd4,Myocd,Hnrnpf,Tnk2,Ano1,Baz2a,Ino80e,Nlrp6,Kcnip2,Ggt1,Zfp809,Clip1,Atp9a,Phf21a,Zc3hc1,Scn8a,Gatad2b,Nsd2,Got1,Ackr1,Mlxip,Ptp4a2,Myof,Piga,Rcbtb2, 1-Mar,Zmym3,Naa25,Usp28,Stk32c,Tbc1d4,Gemin7,Ipo9,Dmrtb1,Plekhj1,Zscan21,Slc39a14,Gsg1,Pex5,Eya1,Pik3r6,Pdlim2,Odf2,Slc9a8,Ldhb,Arhgef9,Vps51,Ip6k2,Cit,Bzw2,Timm23,Sept11,Oas2,Slc6a6,Cbs,Trafd1,Acad10,Six2,Slc35b3,Tmem8b,Gas2l3,Hr,Nudt21,Papola,Adamtsl1,Chtop,Tmed2,Ank3,1110017D15Rik,BC017643,Supt7l,Keap1,Ints7,Crem,Fkbp1a,Ano5,Nlrx1,Slc37a1,Mfge8,Mospd1,Mrps36,Enpp1,Adgre5,Lnpk,Itgam,Naa35,Trim27,Llgl2,Sclt1,Ptpn21,Myo7a,Bcl2l1,Ube2k,Poli,Poc1b,Cpne4,Igsf10,Iscu,Fam126b,Bcas2,Xpo6,Mak,Tarbp2,Kctd15,Kpna1,Sirpa,Dusp27,Mrvi1,Ccnd3,Ikzf4,Bicc1,Ddr2,Acp7,Serpinb6a,Pigb,Mlh3,Mlip,Fn1,Mus81,Ablim2,Atp8b5,Nphp4,Prg4,Mageb16,Cryzl1,Usp8,Prpf39,Ncstn,Klhl18,Ankrd6,Klhl20,Zfp579,Mgme1,Ikbke,Pkn3,Dhps,Dtx3,Sec61a2,Ppp1r13b,Rnf181,Arid3b,Senp8,Gbp3,Sun1,Dbp,Tecr,Lox,Frk,Fgfr3,B3gat1,Atg7,Tshz1,Foxred1,Olfr77,Vps53,Pde1c,Tpm3,Rbm12b2,Spata33,Slc43a2,D130043K22Rik,Orai2,Mid1,Brinp3,Pik3r4,Magi1,Axl,Syt6,Copz2,Ctsd,Adgrb2,Igsf9,Acad8,Pus1,Ppp1r3b,Dusp14,Smim1,Wdr33,Slc2a1,Kcnj15,Bola3,Epha4,Arfip2,Pde8b,Mmp28,Lrrfip1,Vsig10,Bend4,Zfp26,Ppt2,Gpr19,Islr2,Klhl26,Bicdl1,Als2cl,Tcirg1,Gipr,Wdr90,Mcf2,Tyw1,Phf21b,Cept1,Mboat2,Pacsin1,Slc25a13,Ptms,Cnot2,Wnt11,Slc25a53,Kdm2b,Pcnx,Zfp667,Fbxl17,Ptges3,Sox6,Deaf1,Nr1h2,Ikbkg,Lrrc29,Plekha6,Ntsr2,Kmt2d,Fscn1,Slc4a2,Sept2,Fat1,Ptpre,Senp6 |
| **lncRNA** | **9 genes**  ENSMUST00000215759,ENSMUST00000133643,ENSMUST00000148687,ENSMUST00000228670,ENSMUST00000226949,MSTRG.11695.1,ENSMUST00000189960,ENSMUST00000156541,ENSMUST00000124811 | **4 genes**  ENSMUST00000141639,ENSMUST00000142895,ENSMUST00000153113,ENSMUST00000064591 |
| **circRNA** | **202genes**  Nalcn,Lrch3,Agfg1,Gphn,Hecw2,Ash1l,Rnf13,Dmd,Ptprr,Nrxn1,Cacna2d1,Stau2,Focad,Prkce,Tesk2,Sos2,Dgki,Akt3,Elmo1,Slc41a2,Lrba,Ccdc171,Eri3,Fut8,Psd3,Uvrag,Xpo1,Rap1gds1,Atp9b,Vps13b,Cntn5,Tmem132d,Rnf150,Cdk8,Hectd4,Dnm3,Ppp4r1,Chd9,Uap1,Snx7,Rabep1,Alg13,Zeb1,Robo1,Ispd,Trim33,Zmym4,Mllt10,Tenm3,Fam208a,Trim23,Stk39,Dennd1a,Csmd1,Pum1,Ppp1r9a,Ncam2,Kif1b,Kdm1a,Trappc9,Baz2b,Naa35,Itpr2,Atrnl1,Pigk,Tnpo3,Rnf2,Ttc7b,Baz1b,Rere,Ctnna2,Rabgap1l,Mtmr3,Ankib1,Snx29,Yes1,Fam184a,Qser1,Zcchc11,Zfp280c,Ttll5,Sorbs1,Rngtt,Foxn3,Foxj3,Utrn,Dym,Npepps,Smarca1,Dcaf5,Phf8,Kansl1,Cdh19,Tfdp2,Fer,Kdm4cAscc3,Mtcl1,Dlg1,Kcnh1,Med12l,Fam135a,Cbfa2t2,Syne1,Usp32,Cnot1,Mir344g,A330076H08Rik,Ppp2r3a,4930402H24Rik,Fam151b,Taf4b,Rps6kc1,Qk,Fam13b,Rnf214,Myt1l,Ralgapa1,Acvr1b,Eif4e3,Mctp2,Mphosph8,Map4,Ptprd,Mctp1,Fmn1,Abhd2,Zfand4,Vps13a,Bbs9,Ncoa7,Pde8a,Usp54,Ube2cbp,Edil3,Frmpd4,Tmtc4,Phf14,Brd3,Lats1,Lmbr1,Epha6,Zfp638,Slc16a1,Akap10,Strn3,Nbea,Slc4a4,Trp53bp1,Exoc5,Tbc1d5,Sorcs1,Zfyve9,Ankhd1,Cadps,Tdrd3,Adamts6,Smad4,Chd2,Dtna,Tpp2,Arih1,Herc1,Arnt2,Clec16a,Wnk3Sacm1l,Rab31,Phlpp2,Ttc3,Ank3,Vwa8,Bnip2,Nfatc3,Nf1,Cpeb3,Uhrf1bp1l,Lrp1b,Dgke,Ddx10,Yeats2,Srcap,Gm42715,Gpatch2l,Dtnb,Ppa2,Agtpbp1,Iqce,Map2k5,Nek1,Pbrm1,Ate1,Atxn2,Hace1,Kif2a,Ewsr1,Nae1,Rbm33,Afdn,Nvl,Phf3,Diaph2,Hlcs | **123 genes**  Pi4ka,Mtrf1,Arpp21,Stau2,Zfp827,Lrba,Pum1,Gls,Ranbp17Slc4a4,Xpo1,Lrp1b,Ep300,Lrrc49,Akt3,Arhgef9,Myo9a,Nfasc,Fam193a,Cntnap4,Dgki,Nfib,Snx27,Rere,Dennd1b,Dnajc1,Spag9,Atrx,Ano4,Zcchc7,Apbb2,Larp4b,Sorbs1Phkb,Asxl2,Asxl3,Dennd4c,Grm4Sh3bgr,Ehbp1,Ghr,Mindy3,Mctp2,Rsu1,2010111I01Rik,Map2k4,Raph1,Nav1,Ulk2,Ep400,Wdr7,Pik3c2a,Ralgapa1,Mef2d,Rps6kc1,Magi2,Slc9a7,Dcaf6,Rims2,Grk3,Senp5,Cdk12,Ascc3,Rimbp2,Nedd4l,Gmcl1,Fggy,Med12l,Stk39,Clasp2,Rabep1,Cep85l,Fam114a2,Ralgps1,Ilkap,Dpyd,Ice2,Trim37,Trpm7,Ntrk3,Ncoa1,Ano3,Zfr,Ddhd2,Lamp2,AL670603.1,Pik3r3,Slc35e2,Kif16b,Smad1,Mkln1,Ehmt1,Zswim6,Nalcn,Sclt1Csmd1,Smg6,Shq1,Cyld,Atad2b,Arhgap10,Ankrd17,Vav3,Map4k4,Pola1,Zcchc11,Nrxn2,Fmn2,Cadps,Osbpl1a,Slc16a14,Atrnl1,Ttll7,Mdga2,Dync1i1,Itsn2,Ash1l,Hsdl2,Pclo,Ranbp9,Stag1,Nbea,Rasa2 |

**Table S3.** Overlapped genes between pain and Anxiety/Depression related genes.

|  | **TG** | **Sp5C** | **TG&Sp5C** |
| --- | --- | --- | --- |
| **Anxiety** | **85 genes**  SLC6A4,NSD1,NPY,GAL,HNRNPA1,FGFR3,CACNA1G,P2RX7,CALCA,LRRC56,TSC1,WT1,ZEB2,CRH,MTM1,GNAS,SCN8A,FMR1,GLA,OPRK1,ASXL1,KMT2A,GHRL,OPRL1,CCK,NF1,NRAS,SLC2A1,CACNA1A,NOS2,MC1R,PPOX,TCF4,MDH2,FOXP1,HNRNPK,IGF2,SPP1,LIFR,FBN1,RELN,BDNF,BCL7B,DHX30,MAPT,GRIN1,ACE,MME,SCN1B,STXBP1,SCN4A,ATXN2,IRAK1,FGFR1,MEN1,AKT1,KIF1B,MAPK1,KMT2B,ATP1A3,ADORA2A,SCN9A,CLCN6,PTPN11,NGF,CLDN3,CLCN1,KCNA2,NTRK2,TONSL,CTNNB1,USH2A,OPA1,WWOX,UBE3A,CAPN3,LMNA,P2RX4,APC,LIMK1,SCN2A,APOE,POMC,GTF2IRD1,DEAF1 | **58 genes**  SCN9A,CTNNB1,NPPB,POLG,CACNA1A,MLH1,FMR1,FGFR3,FOXP1,GRIA1,NF1,GTF2IRD1,P2RY12,MEN1,NR3C1,PMS1,FBN1,GNAS,IL6,PSENEN,OPRL1,HNRNPK,MME,VIP,IL18,ACE,ATXN2,NTRKa2,FUS,IGF2,KCNQ2,DHX30,TACR1,SLC2A1,NOS2,GNA11,RELN,BRCA2,RAI1,ENG,STAT4,FGFR1,ZEB2,KMT2A,MAPT,IRF5,FKRP,NOS1,OPRM1,CLCN6,DEAF1,TBL2,BDNF,LMX1B,ELN,CHEK2, SCN8A,GTF2I,DLG4 | **30 genes**  FGFR3,ZEB2,GNAS,SCN8A,FMR1,KMT2A,OPRL1,NF1,SLC2A1,CACNA1A,NOS2,FOXP1,HNRNPK,IGF2,FBN1,RELN,BDNF,DHX30,MAPT,ACE,MME,ATXN2,FGFR1,MEN1,SCN9A,CLCN6,NTRK2,CTNNB1,GTF2IRD1,DEAF1 |
| **Depression** | **83 genes**  SLC6A4,NSD1,NPY,GAL,HNRNPA1,ADRA1A,FGFR3,CACNA1G,CDKN1B,P2RX7,ATXN1,CALCA,TSC1,ZEB2,CRH,GNAS,PIGA,FMR1,GLA,GRIA3,ASXL1,KMT2A,GHRL,CCK,NF1,NRAS,SLC2A1,CACNA1A,NOS2,PPOX,EXT2,TCF4,FGF23,IGF2,FBN1,RELN,BDNF,COL1A2,KCNB1,DHX30,NOS3,CACNG2,MAPT,GRIN1,ACE,SCN1B,STXBP1,PANK2,COL11A2,ATXN2,IRAK1,VWF,FGFR1,NEB,MEN1,COL1A1,AKT1,PIK3C2A,MAPK1,ACTB,KMT2B,ATP1A3,ADORA2A,SCN9A,VDR,PTPN11,NGF,CLCN1,NTRK2,TONSL,CTNNB1,USH2A,OPA1,EGF,RYR1,WWOX,LIMK1,SCN2A,APOE,EBF3,POMC,PPARG, DEAF1 | **59 genes**  SCN9A,CTNNB1,NPPB,POLG,CACNA1A,MLH1,FMR1,FGFR3,SLC17A5,GRIA1,NF1,IL13,MEN1,PRKCG,NR3C1,IL1A,FBN1,GRIA3,GNAS,IL6,COL11A1,PSAP,ATRX,CASP3,VIP,IL18,ACE,COL1A2,ATXN2,NTRK2,FUS,IGF2,KCNQ2,VWF,FGFR2,DHX30,TACR1,SLC2A1,NOS2,RELN,BRCA2,RAI1,COL1A1,ALG9,CDKN1B,PIGA,STAT4,FGFR1,ZEB2,KMT2A,MAPT,NOS1,OPRM1,SPR,DEAF1,BDNF,LMX1B,ELN,GTF2I | **29 genes**  FGFR3,CDKN1B,ZEB2,GNAS,PIGA,FMR1,GRIA3,KMT2A,NF1,SLC2A1,CACNA1A,NOS2,IGF2,FBN1,RELN,BDNF,COL1A2,DHX30,MAPT,ACE,ATXN2,VWF,FGFR1,MEN1,COL1A1,SCN9A,NTRK2,CTNNB1,DEAF1 |
| **Anxiety & Depression** | **60 genes**  SLC6A4,NSD1,NPY,GAL,HNRNPA1,FGFR3,CACNA1G,P2RX7,CALCA,TSC1,ZEB2,CRH,GNAS,FMR1,GLA,ASXL1,KMT2A,GHRL,CCK,NF1,NRAS,SLC2A1,CACNA1A,NOS2,PPOX,TCF4,IGF2,FBN1,RELN,BDNF,DHX30,MAPT,GRIN1,ACE,SCN1B,STXBP1,ATXN2,IRAK1,FGFR1,MEN1,AKT1,MAPK1,KMT2B,ATP1A3,ADORA2ASCN9A,PTPN11,NGF,CLCN1,NTRK2,TONSL,CTNNB1,USH2A,OPA1,WWOX,LIMK1,SCN2A,APOE,POMC,DEAF1 | **42 genes**  SCN9A,CTNNB1,NPPB,POLG,CACNA1A,MLH1,FMR1,FGFR3,GRIA1,NF1,MEN1,NR3C1,FBN1,GNAS,IL6,VIP,IL18,ACE,ATXN2,NTRK2,FUS,IGF2,KCNQ2,DHX30,TACR1,SLC2A1,NOS2,RELN,BRCA2,RAI1,STAT4,FGFR1,ZEB2,KMT2A,MAPT,NOS1,OPRM1,DEAF1,BDNF, LMX1B, ELN,GTF2I | **23 genes**  FGFR3,ZEB2,GNAS,FMR1,KMT2A,NF1,SLC2A1,CACNA1A,NOS2,IGF2,FBN1,RELN,BDNF,DHX30,MAPT,ACE,ATXN2,FGFR1,MEN1,SCN9A,NTRK2,CTNNB1,DEAF1 |

**Table S4:** Overlapped genes between pain and Inflammation/Apoptosis/Immunity related genes.

|  | **TG** | **Sp5C** | **TG & Sp5C** |
| --- | --- | --- | --- |
| **Inflammation** | **69 genes**  FGFR,CD8A,CDKN1B,IL6ST,P2RX7,CALCA,LRRC56,WT1,CRH,F8,PRKCD,SERPINC1,CCL3,ALOX5,GHRL,LPIN2,CCK,NF1,HMGB1,CX3CR1,NOS2,TCF4,FGF23,SPP1,FBN1,PSMB4,RELN,BDNF,MAPK14,NOS3,MAPT,ACE,CPT2,MME,BGLAP,IRAK1,VWF,FGFR1,GNE,MVK,COL1A1,AKT1,PIK3C2A,MAPK1,VDR,PRKCQ,PTPN11,NGF,COMP,P4HA2,PRTN3,CTNNB1,EGF,NOD2,TLR1,IL1RAPL2,COL17A1,LMNA,LTA,APC,FN1ACP5,APOE,KRT7,MYH11,POMC,PPARG,CASP8,TCIRG1 | **60 genes**  NFKB1,CTNNB1,NPPB,CASP8,CXCL12,FGFR3,SLC17A5,TCIRG1,NF1,IL13,IL12RB1,NR3C1,IL1A,FBN1,KRT7,P4HA2,IL6,FN1,TGFBR1,CFH,MME,CCR5,CASP3,VIP,IL18,ACE,FLT1,VWF,FGFR2,TACR1,NOS2,BMP6,HMGB1,RELN,BRCA2,CL1A1,EGFR,COL3A1,ENG,CP,CDKN1B,THBD,CAV1,STAT4,CD36,FGFR1,MAPT,IRF5,NOS1,IRF1,LDLR,CD55,PPARA,CDH1,BDNF,ELN,CHEK2,MPZ,ALOX5,ABCB4 | **21 genes**  FGFR3,CDKN1B,ALOX5,NF1,HMGB1,NOS2,FBN1,RELN,BDNF,MAPT,ACE,MME,VWF,FGFR1,COL1A1,P4HA2,CTNNB1,FN1,KRT7,CASP8,TCIRG1 |
| **Apoptosis** | **52 genes**  HNRNPA1,FGFR3,CD8A,CDKN1B,IL6ST,P2RX7,TSC1,WT1,PRKCD,MYB,KMT2A,RARA,ALOX5,HMGB1,STK11,NRAS,CDKN3,NUMA1,NOS2,HNRNPK,IGF2,SPP1,DCC,BDNF,MAPK14,MFN2,NOS3,MAPT,CASP9,MYLK,IRAK1,FGFR1,AKT1,MAPK1,ACTB,VDR,PRKCQPTPN11,NGF,NTRK2,CTNNB1,OPA1,EGF,NOD2,WWOX,LMNA,LTA,APC,FN1,APOE,PPARG,CASP8 | **47 genes**  NFKB1,CTNNB1,CASP8,STAT5B,MLH1,CXCL12,FGFR3,IL13,MYC,PRKCG,NR3C1,IL1A,IL6,IGF2R,FN1,TGFBR1,HNRNPK,PML,CCR5,CASP3,IL18,NTRK2,STK11,FLT1,IGF2,FGFR2,NOS2,BMP6,HMGB1,ACTA2,BRCA2,EGFR,CDKN1B,CAV1,CD36,FGFR1,KMT2A,MAPT,NOS1,ABCC1,IRF1,PPARA,CDH1,BDNF,CHEK2,ABL1,ALOX5 | **16 genes**  FGFR3,CDKN1B,KMT2A,ALOX5,HMGB1,STK11,NOS2,HNRNPK,IGF2,BDNF,MAPT,FGFR1,NTRK2,CTNNB1,FN1,CASP8 |
| **Immunity** | **50 genes**  NSD1,FGFR3,CD8A,IL6ST,P2RX7,LRRC56,F8,PRKCD,CCL3,ALOX5,HMGB1,NRAS,CX3CR1,NOS2,SPP1,LIFR,PSMB4,MAPK14,ACE,MME,IRAK1,VWF,AKT1,MYO5A,MAPK1,ACTB,ADORA2A,VDR,PRKCQ,PTPN11,NGF,TTN,PRTN3,CTNNB1,ACTA1,EGF,NOD2,RYR1,TLR1,LTA,APC,FN1,ACP5,APOE,EPHB4,POMC,PPARG,CASP8,TCIRG1,DEAF1 | **52 genes**  NFKB1,CTNNB1,POLG,CASP8,STAT5B,CXCL12,FGFR3,TCIRG1,IL13,IL12RB1,MYC,NR3C1,IL1A,IL6,FN1,TGFBR1,STIM1,CFH,PML,MME,CCR5,CASP3,VIP,IL18,ACE,VWF,NOS2,SERPING1,BMP6,HMGB1,BRCA2,CD46,EGFR,ENG,CP,THBD,CAV1,STAT4,CD36,IRF5,NOS1,IRF1,DEAF1,CD55,ORAI1,MYO5A,CDH1,LMX1B,CHEK2,MPZ,ABL1,ALOX5 | **12 genes**  FGFR3,ALOX5,HMGB1,NOS2,ACE,MME,VWF,MYO5A,CTNNB1,FN1,TCIRG1,DEAF1 |
| **Inflammation & Apoptosis & Immunity** | **26 genes**  FGFR3,CD8A,IL6ST,P2RX7,PRKCD,ALOX5,HMGB1,NOS2,SPP1,MAPK14,IRAK1,AKT1,MAPK1,VDR,PRKCQ,PTPN11,NGF,CTNNB1,EGF,NOD2,LTA,APC,FN1,APOE,PPARG,CASP8 | **26 genes**  NFKB1,CTNNB1,CASP8,CXCL12,FGFR3,IL13,NR3C1,IL1A,IL6,FN1,TGFBR1,CCR5,CASP3,IL18,NOS2,BMP6,HMGB1,BRCA2,EGFR,CAV1,CD36,NOS1,IRF1,CDH1,CHEK2,ALOX5 | **7genes**  FGFR3,ALOX5,HMGB1,NOS2,CTNNB1,FN1,CASP8 |

**Table S5:** Upregulated pain-related genes, their function and pathway involved.

| **mRNA** | **Description** | **Function** | **Pathway involved** | **Pain related genes in different regions** | |
| --- | --- | --- | --- | --- | --- |
|  |  |  |  | **TG+** | **Sp5C+** |
| Fgfr3 | fibroblast growth factor receptor 3 | angiogenesis, wound healing, cell migration, neural outgrowth | RAS-MAPK pathway, PI3K-AKT pathway, Apoptotic Pathways in Synovial Fibroblasts | 4.1406 | 2.8289 |
| Gnas | GNAS complex locus | activation of adenylyl cyclases and beta-adrenergic receptor | G protein-coupled receptors (GPCRs), Ras signaling pathway | 2.5489 | 2.1571 |
| Fmr1 | Fragile X Messenger Ribonucleoprotein 1 | neuronal development and synaptic plasticity | ATR-dependent signaling pathway | 2.4329 | 2.8498 |
| Slc12a3 | Thiazide-Sensitive Sodium-Chloride Cotransporter | Electroneutral sodium and chloride ion cotransport, Receptor for the pro-inflammatory cytokine IL18 | IL-18 signaling pathway | 2.3751 | 2.1683 |
| Oprl1 | Opioid Related Nociceptin Receptor 1 | receptor for the endogenous, opioid-related neuropeptide, nociception | GPCR downstream signalling, MAP kinases | 1.8783 | 1.8533 |
| Nf1 | Neurofibromin 1 | Stimulates the GTPase activity of Ras | RAS signal transduction pathway | 1.8322 | 2.6439 |
| Cacna1a | Calcium Voltage-Gated Channel Subunit Alpha1 A | involved in muscle contraction and neurotransmitter release | MAPK signaling pathway, all-trans-Retinoic Acid Signaling pathway | 1.6199 | 3.4795 |
| Hnrnpk | Heterogeneous Nuclear Ribonucleoprotein K | neuronal differentiation | EphB-EphrinB Signaling | 1.4408 | 1.2501 |
| Col9a3 | Collagen Type IX Alpha 3 Chain | component of the extracellular matrix in cartilage and intervertebral discs | collagen biosynthesis and modifying enzymes pathway | 1.290084817 | 3.710838385 |
| Fbn1 | fibrillin-1 | structural component of calcium-binding microfibrils | ERK Signaling | 1.242148943 | 2.254593016 |

**Table S6:** Downregulated pain-related genes, their function and pathway involved.

| **mRNA** | **Description** | **Function** | **Pathway involved** | **Pain related genes in different regions** | |
| --- | --- | --- | --- | --- | --- |
|  |  |  |  | **TG-** | **Sp5C-** |
| Deaf1 | deformed epidermal autoregulatory factor 1 | development, differentiation, proliferation, and apoptosis | Sudden infant death syndrome (SIDS) susceptibility pathways | -5.6269 | -2.6141 |
| Lox | Lysyl Oxidase | Regulator of Ras expression, tumor suppression | Canonical and non-canonical TGF-B signaling, | -3.2641 | -1.5054 |
| Enpp1 | Ectonucleotide Pyrophosphatase/Phosphodiesterase 1 | bone mineralization and soft tissue calcification | Endochondral ossification and Insulin signaling | -2.4719 | -1.9405 |
| Prx | Periaxin | transmission of nerve impulses and normal perception of sensory stimuli | EGR2 and SOX10-mediated initiation of Schwann cell myelination | -1.9548 | -3.3579 |
| Col1a1 | Collagen Type I Alpha 1 Chain | formation and maintenance of bones, skin, tendons, and ligaments | Angiotensin II receptor type 1 pathway | -1.2427 | -1.4887 |
| Vwf | Von Willebrand Factor | formation of a stable blood clot | PI3K-Akt-mTOR-signaling pathway | -1.1890 | -1.1103 |
| Mapt | Microtubule Associated Protein Tau | maintenance of neuronal polarity | AMPK Signaling Pathway P38 MAPK Signaling Pathway | -1.0677 | -2.2332 |
